# Galectin-3 mediates lysosome-related inflammation within monocyte-derived macrophages in a mouse model of ischemic brain injury

**DOI:** 10.1172/JCI194139

**Published:** 2026-02-17

**Authors:** Miao Wang, Zhentai Huang, Zhihong Du, Jiajing Shan, Qing Ye, Lingxiao Lu, Ming Jiang, Fei Xu, Ziyang Liu, David J.R. Fulton, Rehana K. Leak, Babak Razani, Jun Chen, Xiaoming Hu

**Affiliations:** 1 Geriatric Research Education and Clinical Center, Veterans Affairs Pittsburgh Health Care System, Pittsburgh, Pennsylvania, USA.; 2 Department of Neurology, University of Pittsburgh School of Medicine, Pittsburgh, Pennsylvania, USA.; 3 Department of Medicine and Vascular Medicine Institute, University of Pittsburgh School of Medicine, University of Pittsburgh Medical Center, Pittsburgh, Pennsylvania, USA.; 4 Vascular Biology Center, Medical College of Georgia, Augusta University, Augusta, Georgia, USA.; 5 Division of Pharmaceutical Sciences, Duquesne University, Pittsburgh, Pennsylvania, USA.; 6 Pittsburgh VA Medical Center, Pittsburgh, Pennsylvania, USA.

**Keywords:** Inflammation, Neuroscience, Stroke

## Abstract

Circulating monocyte-derived macrophages (MDMø) rapidly invade the brain after stroke, exerting both detrimental and beneficial effects. Elucidating mechanisms that mediate detrimental properties of MDMø may identify therapeutic strategies to divert MDMø from destructive phenotypes, while preserving their favorable effects. Toward this goal, the current study explores the function of Galectin-3 (GAL3) in MDMø and elucidates mechanisms whereby MDMø-derived GAL3 exacerbates stroke injury. In the acutely injured brain, GAL3 expression was upregulated primarily within MDMø. Global KO of GAL3 reduced brain infarcts in the short term but did not sustain long-term positive outcomes. Using BM chimera mice, macrophage transplantation, and myeloid cell–specific GAL3-KO (*LysM*^Cre+/–^*Lgals3*^fl/fl^) mice, we demonstrated that GAL3 in MDMø mediated acute infarct expansion after stroke. Coculturing brain lysate–treated, BM-derived macrophages (BMDMs) with oxygen glucose deprivation–challenged neurons induced neurotoxicity that was mitigated by the cell-permeable, selective GAL3 inhibitor TD139. GAL3 triggered cathepsin induction and lysosomal leakage in BMDMs, leading to inflammasome activation. Systemic and transient TD139 treatment in the acute injury phase reduced infarcts, tempered neuroinflammation, and improved long-term neurological outcomes. Therefore, MDMø-derived GAL3 represents a drug target that could be accessed in peripheral blood to potentially mitigate post-stroke brain injury.

## Abstract 2

We investigate whether high galectin-3 expression mediates harmful responses within monocyte-derived macrophages after stroke, triggering leakage of lysosomal contents, exacerbating neuroinflammation, and worsening cerebral infarcts.

## Graphical abstract

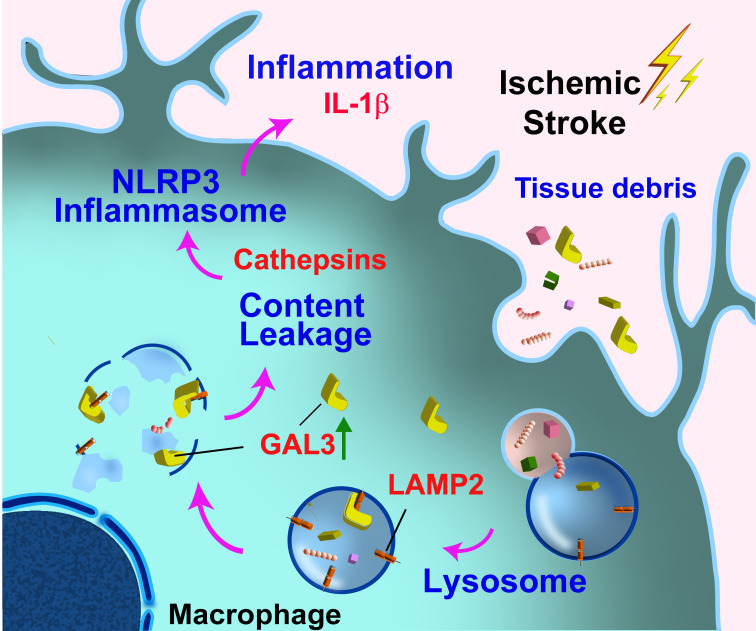

## Introduction

Ischemic stroke results in rapid cell death in the core region of blood flow reduction. This acute-stage cell death is followed by cerebral and systemic immune responses that drive the progression of brain injury but also promote the repair of brain tissues. Hence, targeting early inflammatory responses after stroke may profoundly influence stroke outcomes and represent a promising avenue for clinical treatment.

Blood monocytes are among the fastest responders to cerebral ischemia. Monocytes permeate the injured brain in a CCR2-dependent manner and give rise to brain monocyte-derived macrophages (MDMø) (1). The number of inflammatory monocytes increases in circulation as early as 3 h after stroke onset. Significant infiltration of MDMø is observed in the ischemic brain 1 d after stroke, with a peak at 3 d and an elevation for at least 28 d (2). Interestingly, MDMø appear to infiltrate the brain as a LY6C^hi^CCR2^+^ classically activated inflammatory phenotype in the acute/subacute stages of stroke and then switch to an antiinflammatory phenotype in the chronic injury phase (2, 3). Accordingly, MDMø play diverse roles at different stages of stroke. CCR2 deficiency prevents MDMø infiltration and reduces brain infarcts early after stroke (4) but impairs long-term brain repair and recovery (5). Adding to the complexities of MDMø function are the protective effects of inflammatory MDMø early after stroke. For example, MDMø are important for phagocytic clearance of debris early after stroke (6). Thus, elucidating mechanisms that mediate detrimental properties of MDMø early after stroke may identify specific targeting strategies to divert MDMø from a destructive phenotype while preserving their favorable effects. Transient application of these strategies could leave the late-phase brain repair properties of MDMø undisturbed and exert net beneficial effects on the function of the CNS.

Galectin-3 (GAL3) is a carbohydrate-binding protein expressed in myeloid cells and other cell types (7). Studies in stroke patients consistently show a link between higher blood levels of GAL3 within the acute stage (hours to days after stroke) and poor outcomes or higher mortality (8). A persistent increase in GAL3 expression, mainly within microglia and infiltrating MDMø, is observed in ischemic brains after experimental stroke (9, 10). Studies on the functions of GAL3 in stroke and other CNS injury models have yielded contradictory data, as both detrimental (11–13) and beneficial (14–16) effects of GAL3 have been reported early after brain ischemia. In addition, GAL3 promotes angiogenesis for brain repair (10, 16, 17), revealing that GAL3 plays diverse, temporally divergent, context-dependent, and cell-specific roles in the injured CNS (7). Some studies showed that GAL3 deficiency exerts no impact on stroke outcomes, perhaps because the opposing effects of GAL3 negate each other (17, 18). Notably, all prior work in this area relied on global GAL3-KO mice or direct injection of GAL3 or GAL3 inhibitors into the CNS, failing to distinguish the effects of GAL3 in microglia versus infiltrating MDMø. Therefore, a dissection of the cell-specific functions of GAL3 in MDMø is necessary.

The current study elucidates a previously undefined mechanism by which upregulation of GAL3 in MDMø exacerbates acute brain injury after stroke. High GAL3 expression in MDMø increases lysosomal cathepsin B expression and leakage, which exacerbates neuroinflammation and worsens outcomes. Systemic and transient GAL3 inhibition early after stroke therefore reduces brain infarction, tempers neuroinflammation, and improves neurological functions in both young and aged mice. Given the accessibility of peripheral monocytes for therapeutic manipulation, targeting MDMø-derived GAL3 may represent a treatment that could be readily translated into clinical practice for stroke patients.

## Results

### GAL3 expression is rapidly increased in brain-invading MDMø after ischemic stroke.

**Figure 1:**
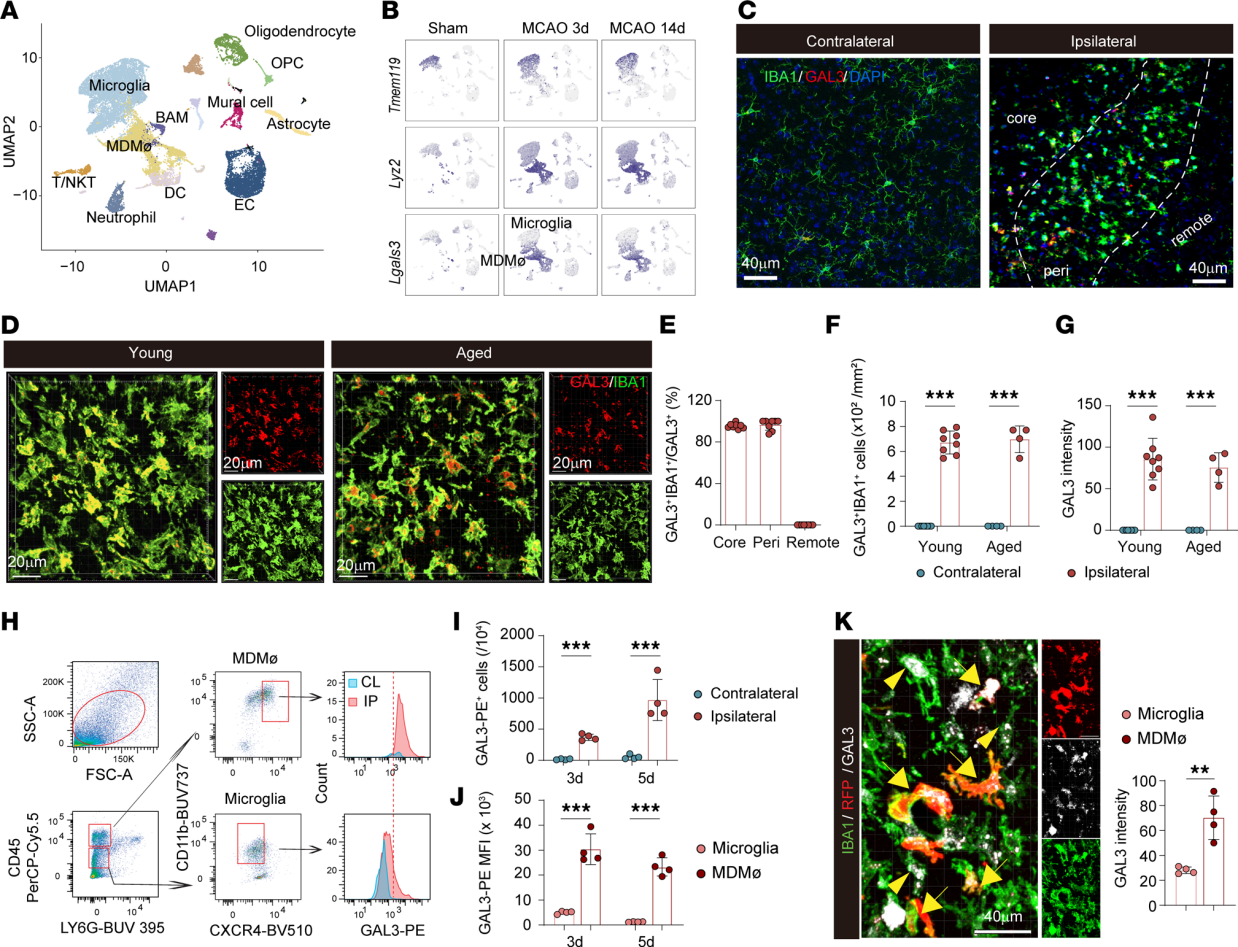
GAL3 expression is rapidly increased in brain-invading MDMø after ischemic stroke. (**A**) UMAP plot showing clustering and cluster annotations of brain cells from young male mice 3 and 14 d after tMCAO or sham operation. BAM, border-associated Mø; EC, endothelial cells; OPC, oligodendrocyte precursor cells; T/NKT, T cells and natural killer T cells. (**B**) UMAP plots illustrating the expression of signature genes for microglia (*Tmem119*) and MDMø (*Lyz2*) and demonstrating the expression of *Lgals3* in MDMø and microglia. (**C**) Representative double labeling of IBA1 and GAL3 in ischemic brain and contralesional brain tissues 5 d after tMCAO. Scale bars: 40 μm. (**D**) Immunostaining of IBA1 and GAL3 in peri-infarct areas of young (5-month-old) and aged (20-month-old) mice 5 d after tMCAO. Scale bars: 20 μm. (**E**) Percentage of GAL3^+^IBA1^+^cells among all GAL3^+^ cells in ischemic core, peri-infarct (peri), and remote zones in the ischemic brain. (**F**) Numbers of GAL3^+^IBA1^+^ cells in the ischemic core and peri-infarct areas. (**G**) Quantification of GAL3 intensity within IBA1^+^ cells in the ischemic core and peri-infarct areas. *N* = 4–8/group for **E**–**G**. (**H**) Flow cytometry to detect GAL3 expression in CD45^hi^LY6G^−^CD11b^+^CXCR4^+^ MDMø and CD45^int^LY6G^−^CD11b^+^CXCR4^−^ microglia in ischemic brains after tMCAO. (**I**) Number of total GAL3^+^CD45^+^CD11b^+^LY6G^−^ cells in ipsilateral and contralateral brains of young mice 3 and 5 d after tMCAO. (**J**) MFI of GAL3 in MDMø and microglia. *N* = 4/group for **I** and **J**. (**K**) Left: Representative GAL3 and IBA1 staining in peri-infarct area of young *Ccr2*^CreER^Ai14(RFP) mice 5 d after tMCAO. Arrows mark GAL3^+^IBA1^+^CCR2(RFP)^+^ infiltrating MDMø. Arrowheads mark GAL3^+^IBA1^+^RFP^–^ microglia. Right: Quantification of GAL3 intensity in RFP^+^IBA1^+^ MDMø and RFP^–^IBA1^+^ microglia. *N* = 4/group. **P < 0.01, ***P < 0.001. Two-tailed, unpaired Student’s *t* test (**K**) or 2-way ANOVA and Bonferroni’s test (**F**, **G**, **I**, and **J**).

We analyzed scRNA-seq datasets from different publications (3, 19) to define the cellular expression of *Lgals3*, the gene encoding GAL3, in the ischemic brain. In the first dataset (19), *Lgals3* was barely detected in the sham brain but was highly expressed in MDMø (identified by high expression of *Lyz2* and low expression of *Tmem119*) and expressed at a lower level in microglia (identified by high expression of *Tmem119* and low expression of *Lyz2*) at both 3 and 14 d after transient middle cerebral artery occlusion (tMCAO) for 60 min (Figure 1, A and B). Analysis of another single-cell transcriptomic study of brain and blood cells collected 2 or 14 d after tMCAO (GSE225948) (3) revealed a similar distribution of *Lgals3* in sorted CD45^hi^ cells of the brain (Supplemental Figure 1, A and B; supplemental material available online with this article; https://doi.org/10.1172/JCI194139DS1) as well as high *Lgals3* expression in peripheral monocytes and neutrophils (Supplemental Figure 1, C and D). Immunostaining verified the low basal expression of GAL3 in the contralateral unlesioned brains, while its expression increased in the core and peri-infarct areas of ischemic brains (Figure 1, C and D). Almost all GAL3^+^ signal was detected in IBA1^+^ MDMø/microglia (Figure 1E). High GAL3^+^IBA1^+^ cell counts (Figure 1F) and robust GAL3 signal within IBA1^+^ cells (Figure 1G) were detected in the ischemic core and peri-infarct areas of both young and aged male mice 5 d after tMCAO. GAL3 expression was also detected in some IBA1^+^CD206^+^ border-associated Mø, although the number of border-associated Mø was low (Supplemental Figure 1G). GAL3 was not expressed in MPO^+^ infiltrating neutrophils (Supplemental Figure 1E) and was expressed at low levels in CD11c^+^ DCs (Supplemental Figure 1F), consistent with scRNA-seq data (Figure 1, A and B, and Supplemental Figure 1, A and B). GAL3 signal was nearly undetectable in other CNS cells, such as NEUN^+^ neurons (Supplemental Figure 1H), CD31^+^ endothelial cells (Supplemental Figure 1I), or GFAP^+^ astrocytes (Supplemental Figure 1J).

We used flow cytometry to distinguish GAL3 expression in MDMø versus microglia 3 and 5 d after tMCAO. MDMø and microglia were distinguished by CD45 and CXCR4, a marker of hematopoietic stem cell–derived monocytes/Mø (Figure 1H) (20). An increased number of GAL3^+^CD45^+^CD11b^+^LY6G^−^ MDMø/microglia were observed in the ischemic hemisphere compared with the contralateral hemisphere (Figure 1I). In addition, the intensity of the GAL3 signal was considerably higher in CD45^hi^CD11b^+^LY6G^−^CXCR4^+^ MDMø compared with CD45^int^CD11b^+^LY6G^−^CXCR4^−^ microglia (Figure 1J). Similar results were obtained using *Ccr*2^CreER^Ai14 (RFP) reporter mice, in which infiltrating CCR2(RFP)^+^IBA1^+^ MDMø and CCR2^−^IBA1^+^ microglia were observed in core and peri-infarct areas (Figure 1K). Similarly, GAL3 expression was distinctly higher in CCR2^+^IBA1^+^ MDMø than in CCR2^–^IBA1^+^ microglia (Figure 1K).

### Global GAL3 KO diminishes brain infarcts early after stroke but does not sustain long-term positive outcomes.

**Figure 2:**
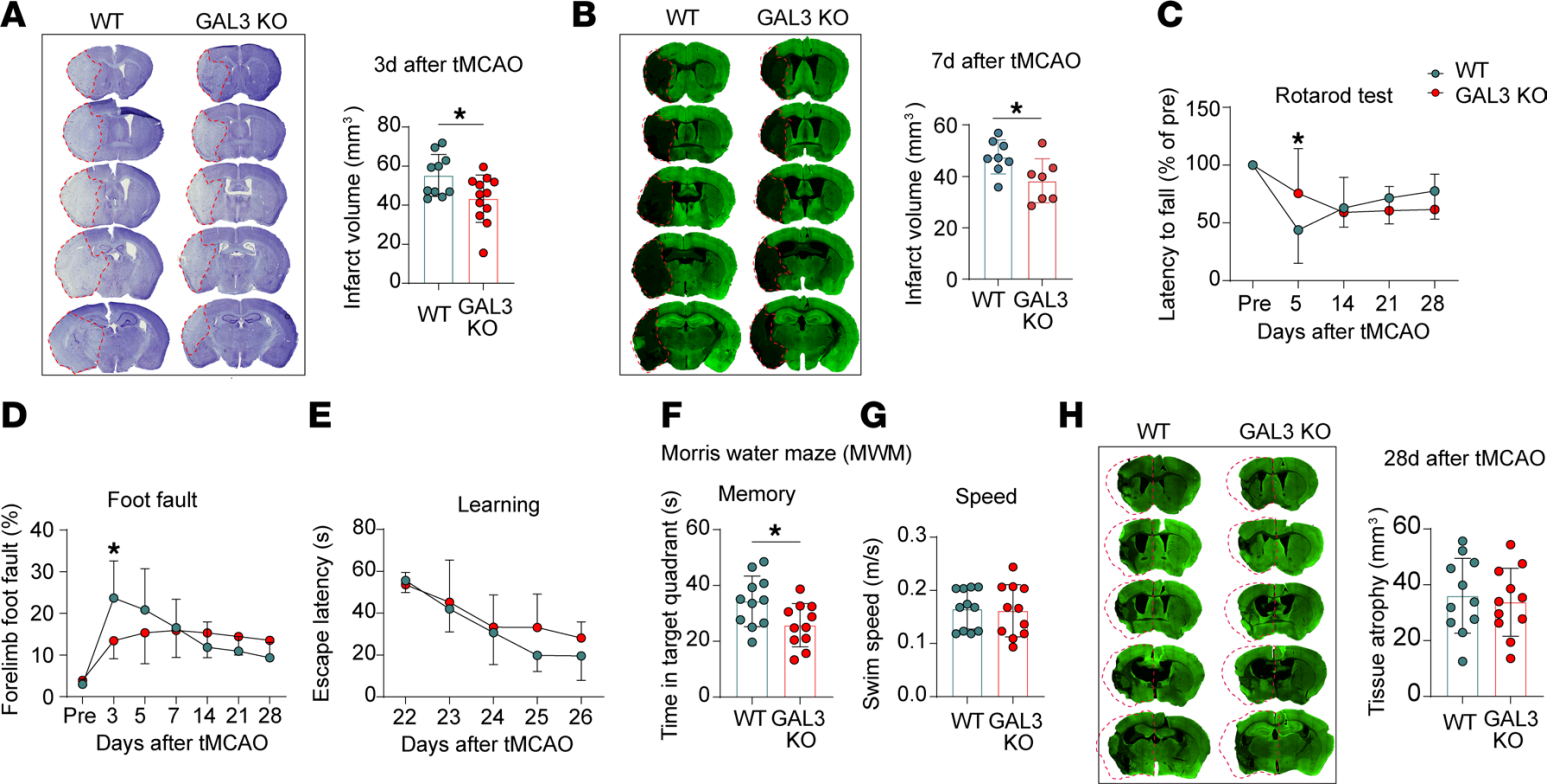
GAL3 KO reduces brain infarct in the early injury phase but does not improve neurological outcomes in the chronic injury phase after stroke. WT and GAL3-KO mice were subjected to 60 min tMCAO. (**A**) Representative Nissl staining and quantification of brain infarct 3 d after tMCAO. *N* = 10–12/group. The red dashed lines trace the border of infarct lesion. (**B**) Representative MAP2 staining and quantification of brain atrophy 7 d after tMCAO. *N* = 7–8/group. The red dashed lines trace the border of infarct lesion. (**C** and **D**) Sensorimotor function was assessed using the rotarod (**C**) and foot fault (**D**) tests. (**E**–**G**) Cognitive function was assessed using the Morris water maze test. (**E**) The latencies to locate the escape platform during the learning phase. (**F**) Time spent in the target quadrant where the escape platform was previously located during the memory probe test. (**G**) Average swim speed across the test phase. (**H**) Representative MAP2 staining and quantification of brain atrophy 28 d after tMCAO. The areas of contralesional hemisphere are reflected on the ipsilesional hemisphere (red dashed lines). *N* = 11/group for **C**–**H**. *P < 0.05. Two-tailed, unpaired Student’s *t* test (**A**, **B**, **F**, **G**, and **H**) and 2-way repeated measures ANOVA and Bonferroni’s test (**C**–**E**).

First, we used *Lgals3* global KO mice to assess the importance of GAL3 in short- and long-term stroke outcomes. GAL3 deficiency reduced brain infarct volume 3 and 7 d after tMCAO (Figure 2, A and B). Consistent with lower ischemic lesion sizes at days 3 and 7, GAL3-KO mice demonstrated improved sensorimotor functions within a week after tMCAO, as shown by longer retention times on a rotating bar (Figure 2C) and fewer foot faults (Figure 2D). However, this short-term protection was unsustainable. Differences in sensorimotor functions between the 2 genotypes waned over time, as behavioral performance improved over time in WT but not GAL3 KO mice (Figure 2, C and D). Moreover, GAL3-KO mice displayed worsened memory deficits (Figure 2F) in the Morris water maze compared with WT mice at 27 d after tMCAO, despite their comparable learning capacity (Figure 2E) and swim speeds (Figure 2G). GAL3-KO and WT mice showed similar brain atrophy 28 d after tMCAO (Figure 2H). Collectively, our data suggest that high GAL3 expression exacerbates acute ischemic brain injury, although the continued expression of GAL3 is essential for long-term recovery after stroke.

### High GAL3 expression in MDMø mediates infarct expansion early after stroke.

**Figure 3:**
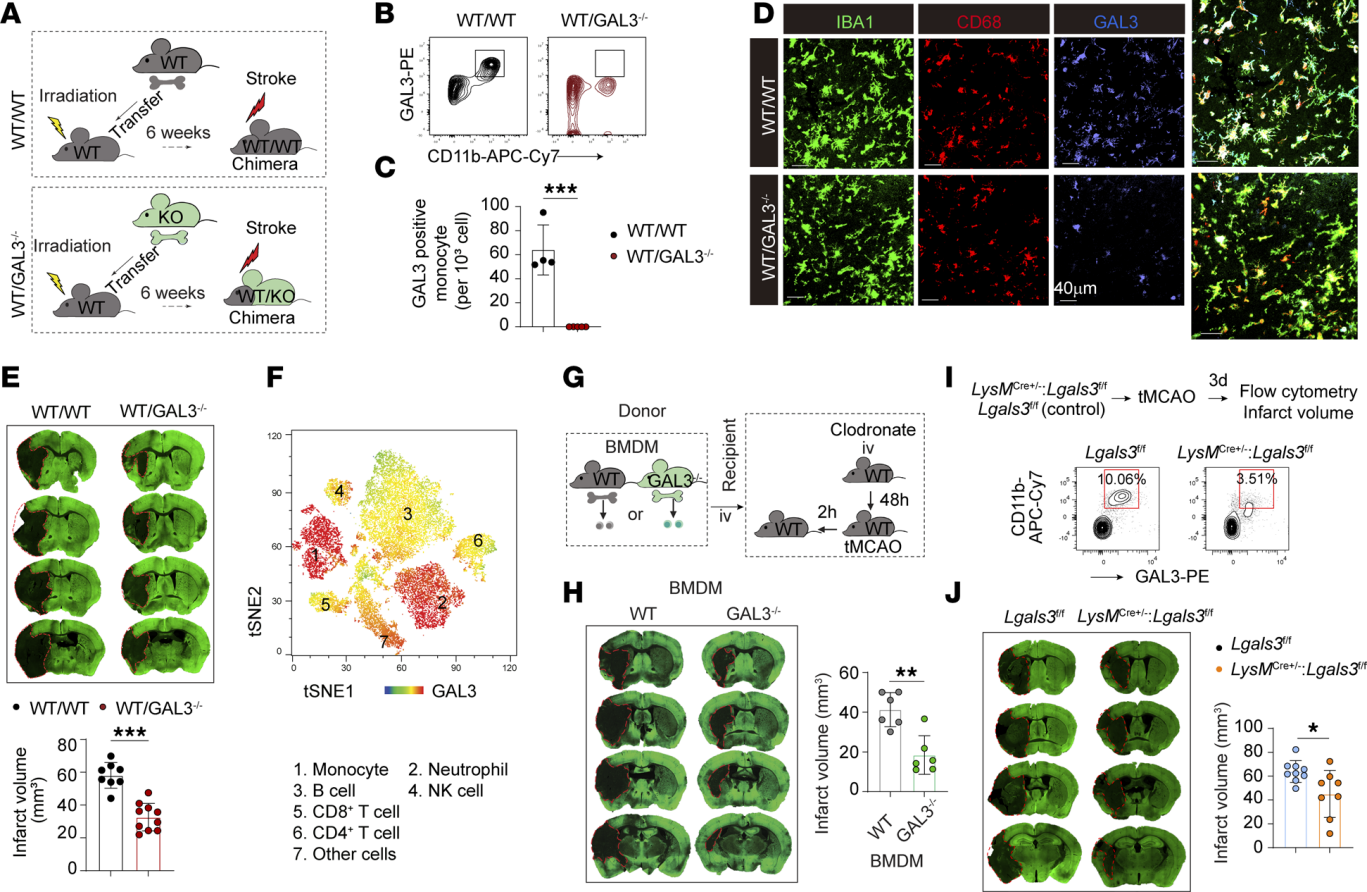
GAL3 expression in monocyte/Mø is instrumental in infarct expansion in the early stroke injury phase. (**A**) Experimental design of BM transplantation. (**B** and **C**) Flow cytometry detected depletion of GAL3^+^CD11b^+^ monocytes in the blood of WT/GAL3^–/–^ chimera mice but not in WT/WT chimera mice. *N* = 4–5/group. (**D**) IBA1/CD68/GAL3 staining in ischemic brains from BM chimera mice 3 d after tMCAO. Scale bar: 40 µm. (**E**) Brain infarct was measured in WT/GAL3^–/–^ and WT/WT chimera mice by MAP2 staining 3 d after tMCAO. *N* = 8–10/group. (**F**) Flow cytometry detection of GAL3 expression in different types of blood immune cells in WT mice 3 d after tMCAO. (**G**) Experimental design of monocyte depletion and BMDM adoptive transfer. (**H**) Brain infarcts were measured 3 d after after tMCAO by MAP2 staining in mice transferred with WT or GAL3^–/–^ BMDMs. *N* = 6/group. (**I**) Myeloid cell–specific *Lgals3-*KO mice (*LysM*^cre+/–^*Lgals3*^fl/fl^) or control mice (*Lgals3*^fl/fl^) were subjected to 60 min tMCAO. Flow cytometry confirmed reduction of GAL3^+^CD11b^+^LY6G^−^ monocytes in the blood of *LysM*^cre+/–^*Lgals3*^fl/fl^ mice compared with *Lgals3*^fl/fl^ mice. (**J**) Brain infarct was measured by MAP2 staining 3 d after tMCAO. *N* = 8–9/group. *P < 0.05, **P < 0.01, ***P < 0.001. Two-tailed, unpaired Student’s *t* test.

We explored whether the high expression of GAL3 in MDMø early after stroke underlies its detrimental effects on early stroke outcomes. To achieve GAL3 deletion in monocytes and MDMø without affecting GAL3 in microglia, BM chimera mice were constructed using our established protocol (21). The BM of GAL3-KO or WT mice was transferred to irradiated WT recipients (Figure 3A). WT/GAL3-KO chimera and WT/WT chimera were subjected to tMCAO 6 weeks after BM transplantation. WT/GAL3-KO chimera and WT/WT chimera mice displayed similar immune cell compositions within blood (Supplemental Figure 2, A and B). We confirmed that expression of GAL3 was abolished in CD11b^+^LY6G^−^ monocytes in blood (Figure 3, B and C). We also capitalized on CD68 as a marker of phagocytic macrophages and observed dramatic suppression of IBA1^+^CD68^+^GAL3^+^ MDMø in the peri-infarct areas in the WT/GAL3-KO chimera 3 d after tMCAO (Figure 3D), consistent with our earlier findings that GAL3 signal in mononuclear phagocytes was largely of MDMø and not microglial origin (see Figure 1, J and K). WT/GAL3-KO mice also exhibited lower brain lesion sizes 3 d after stroke compared with WT/WT mice (Figure 3E). To exclude the possible effect of irradiation on resident microglia, we performed a separate set of experiments constructing WT/WT and WT/GAL3 chimera mice, with the recipients’ heads shielded during irradiation (Supplemental Figure 2C). Similarly, the WT/GAL3-KO mice exhibited lower brain lesion sizes 3 d after stroke compared with WT/WT mice (Supplemental Figure 2D).

While evaluating GAL3 expression in WT blood cells, we discovered high expression in both CD11b^+^LY6G^−^ monocytes and CD11b^+^LY6G^+^ neutrophils (Figure 3F). To further explore the function of GAL3 in MDMø, we performed Mø transfer experiments. First, WT mice were injected intravenously with clodronate liposomes to achieve monocyte/Mø depletion 48 h before 60 min tMCAO. BM-derived Mø (BMDMs) were prepared from the WT and GAL3-KO mice as previously described (21). BMDMs were injected intravenously (2 million/mouse) into monocyte/Mø-depleted mice 2 h after tMCAO (Figure 3G), and mice were euthanized 3 d after stroke. Mice injected with GAL3-KO BMDMs showed smaller infarcts compared with mice with WT BMDM injections (Figure 3H). We then performed WT or GAL3-KO BMDM transfer into Rag1-KO lymphopenic recipients, following the same experimental procedure (Supplemental Figure 2E). Similarly, the infarct volume was smaller in Rag1-KO mice with GAL3-KO BMDM transfer, compared with those with WT BMDM transfer (Supplemental Figure 2F). These data suggest that the presence of lymphocytes is not essential for the effect of GAL3 in MDMø.

Finally, we constructed Mø-specific GAL3-KO mice (*LysM*^cre+/–^*Lgals3*^fl/fl^) by crossing the *LysM*^cre+/–^ mice with *Lgals3*^fl/fl^ mice (Figure 3I). Flow cytometry confirmed a reduction in the number of GAL3^+^CD11b^+^ monocytes/Mø in *LysM*^cre+/–^*Lgals3*^fl/fl^ mice 3 d after tMCAO (Figure 3I). The cell-specific KO of GAL3 in monocytes/Mø reduced brain lesion size 3 d after tMCAO compared with *LysM*^cre+/–^ controls (Figure 3J). Collectively, these data demonstrate that the dramatic post-stroke increase in GAL3 expression within MDMø exacerbates ischemic brain injury in the acute phase.

### GAL3 accumulates rapidly in the lysosomes of MDMø after acute ischemic brain injury.

**Figure 4:**
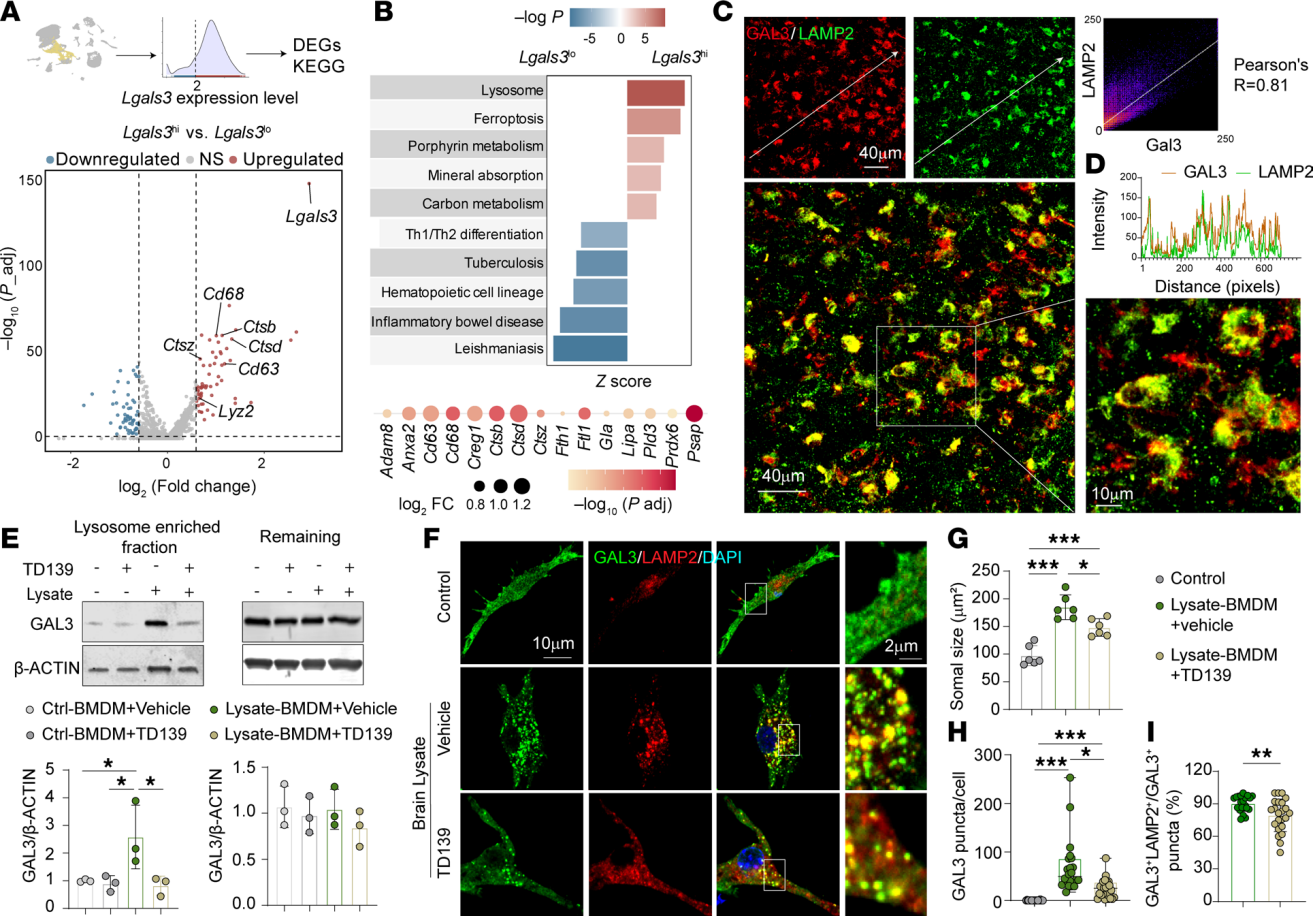
GAL3 accumulates rapidly in the lysosomes of MDMø after acute ischemic brain injury. (**A**) Experimental scheme illustrating the analysis of differentially expressed genes (DEGs) between *Lgals3*^hi^ and *Lgals3*^lo^ MDMø 3 d after tMCAO. MDMø were divided into 2 groups based on *Lgals3* expression level > 2 or ≤ 2. DEGs were identified by comparing the 2 groups. A volcano plot depicts the DEGs [log (fold change) > 0.59 or < –0.59, Bonferroni adjusted *P* value < 0.05] between *Lgals3*^hi^ and *Lgals3*^lo^ MDMø. (**B**) KEGG enrichment analysis was performed on the DEGs using Metascape. Shown are the 10 KEGG pathways predicted to be upregulated or downregulated (*z* score ≥ 5 or *z* score ≤ 5). Corresponding dot plot shows upregulated genes involved in the lysosome term in the *Lgals3*^hi^ group. (**C**) Representative LAMP2 and GAL3 staining in ischemic brains 3 d after tMCAO. Right panel shows Pearson’s correlation analysis of GAL3 and LAMP2 signal. Scale bars: 40 μm, 10 μm (zoom). (**D**) Fluorescence intensity profiles of GAL3 (red) and LAMP2 (green) signal for objects crossing the white arrows in **C**. (**E**) Immunoblot and quantitative analysis of GAL3 expression in lysosome-enriched fraction and the remaining cell lysates (without lysosome) from cultured BMDMs under the indicated conditions. *N* = 3/condition. (**F**) Representative images of GAL3 and LAMP2 staining in control and brain lysate–treated BMDMs with or without TD139 (10 μM). (**G**) The average somal size of BMDMs. *N* = 6 per condition. (**H**) The numbers of GAL3^+^ puncta per cell. (**I**) The percentages of GAL3^+^LAMP2^+^ puncta among total GAL3^+^ puncta under indicated conditions. *N* = 22–23 randomly selected cells per condition from 3 independent experiments. *P < 0.05, **P < 0.01; ***P < 0.001. Two-tailed, unpaired Student’s *t* test (**I**), 1-way ANOVA and Bonferroni’s test (**E** and **G**), or Kruskal-Wallis test (**H**).

We analyzed differentially expressed genes between *Lgals3*^hi^ MDMø and *Lgals3*^lo^ MDMø 3 d after tMCAO (Figure 4A). Numerous lysosome-related genes, including *Cd68*, *Ctsb*, *Ctsz*, and *Lyz2*, were upregulated in *Lgals3*^hi^ MDMø. Kyoto Encyclopedia of Genes and Genomes (KEGG) analyses identified the upregulation of pathways related to lysosomes and ferroptosis (Figure 4B). Consistent with the functional association of GAL3 with lysosomal damage in phagocytes (22, 23), the distribution of *Lgals3* signal in the brain overlapped with *LAMP2*, a marker of lysosomes, and the colocalized signal was distributed mainly within the MDMø population as expected (Supplemental Figure 3, A and B). GAL3^+^LAMP2^+^ puncta within IBA1^+^ cells were observed in peri-infarct areas 3 d after tMCAO (Supplemental Figure 3C). Indeed, more than 97% of GAL3^+^IBA1^+^ cells were double stained with LAMP2 (Supplemental Figure 3D), and the GAL3 signal was highly colocalized with LAMP2 (Figure 4, C and D). This relatively close association between GAL3 and LAMP2 signal was reflected in the high Pearson’s correlation coefficient (*r* = 0.81) of the 2 types of immunopositive signal (Figure 4C).

We then used in vitro BMDM cultures to assess the elevation of GAL3 in BMDMs upon exposure to brain lysates and the spatial association of GAL3 with lysosomes. Brain lysates were prepared from WT brain as described (24). BMDMs prepared from WT mice were exposed to brain lysates for 6 h, with or without TD139, a selective and cell-permeable GAL3 inhibitor. We measured GAL3 expression in crude lysosome fractions using immunoblotting. Exposure to brain lysates increased GAL3 expression in the lysosome fraction, and this effect was inhibited by TD139 (Figure 4E). The expression of GAL3 in the remaining cellular fraction (i.e., excluding lysosomes) showed no significant changes (Figure 4E). Therefore, TD139 reduced the total expression of GAL3 in activated BMDMs. This is consistent with a previous study showing that inhaled TD139 suppresses GAL3 expression in alveolar Mø (25). Similar results were achieved when BMDMs were treated with myelin fragments. Exposure to myelin fragments at 0.6 to 1.2 μg slightly increased GAL3 expression in total protein from BMDMs (Supplemental Figure 4A). Importantly, 0.6 μg myelin greatly elevated GAL3 expression in the crude lysosome fraction from BMDMs, and this effect was similarly inhibited by TD139 (Supplemental Figure 4B). Exposure to dead neurons also increased GAL3 immunostaining in BMDMs, which was colocalized with LAMP2 signal as expected, particularly in perinuclear locations (Supplemental Figure 4C).

Immunostaining showed that brain lysate exposure resulted in activation of BMDMs, as revealed by an increase in somal size, and this hypertrophy was partially reversed by TD139 (Figure 4, F and G). The number of GAL3^+^ puncta also increased significantly in BMDMs after encountering brain lysates, with TD139 inhibiting this elevation (Figure 4H). Nearly 90% of GAL3^+^ puncta were GAL3^+^LAMP2^+^ in vehicle-treated BMDMs, but TD139 lowered the percentages of GAL3^+^LAMP2^+^ puncta (Figure 4I). Further analyses of lysosome morphology and subcellular positioning revealed that, whereas lysosomes were evenly distributed in untreated control BMDMs, brain lysate exposures increased lysosomal clustering in the perinuclear region, and this morphological effect was corrected by TD139 treatment (Supplemental Figure 4D). The diameter and area of lysosomal puncta also increased in BMDMs after brain lysate exposure, especially in the perinuclear area (>2.4 μm from cell edge), and this increase was similarly ameliorated by TD139 (Supplemental Figure 4, E and F).

These data demonstrate that GAL3 accumulates within lysosomes after exposures of MDMø to noxious stimuli, increasing perinuclear lysosomal sizes and shifting their spatial distribution.

### GAL3 induces a neurotoxic phenotype in MDMø.

**Figure 5:**
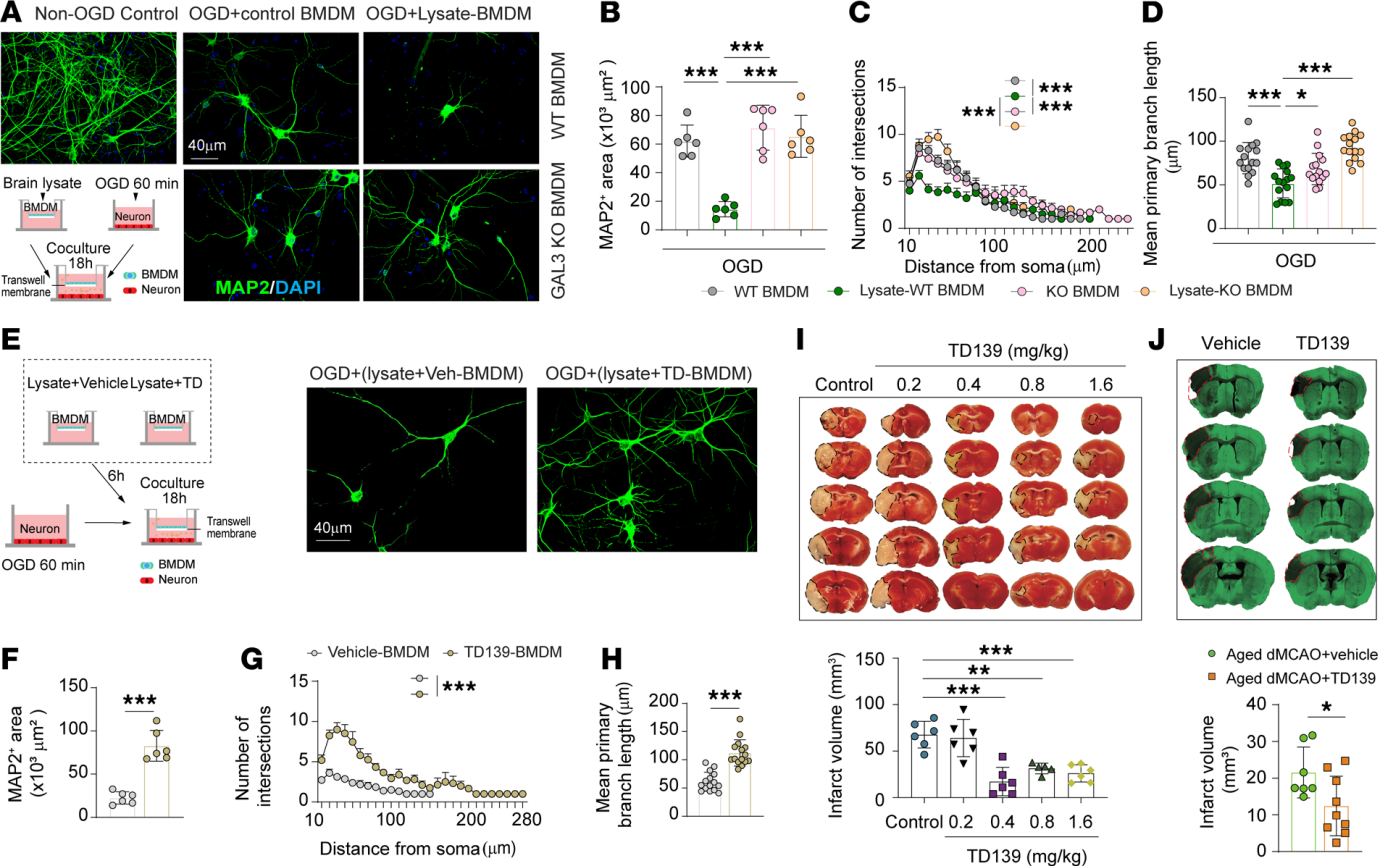
GAL3 induces a neurotoxic phenotype in MDMø. (**A**) The schematic shows the experimental design. Representative images show MAP2 neuronal immunostaining in cocultures. Scale bar: 40 μm. (**B**) The coverage areas of MAP2-stained neurons were quantified. *N* = 6/condition. (**C** and **D**) Sholl analysis displaying dendritic complexity. (**C**) Number of intersections plotted against distance from the cell soma. (**D**) Mean primary branch length. *N* = 15 neurons randomly selected from 3 independent experiments. (**E**) BMDMs in culture inserts were treated with brain lysates and TD139 (10 μM) or vehicle for 6 h. The treated BMDMs were then cocultured with OGD neurons for 18 h. Representative images show MAP2 immunostaining under indicated conditions. Scale bar: 40 μm. (**F**) Coverage areas of MAP2-stained neurons. *N* = 6/condition. (**G** and **H**) Sholl analysis displaying dendritic complexity. *N* = 15 randomly selected neurons from 3 independent experiments. (**G**) Number of intersections plotted against distance from the cell soma. (**H**) Mean primary branch length. (**I**) Young male mice were subjected to 60 min tMCAO and treated with different doses of TD139 (starting 2 h after tMCAO, then daily for 2 d). Infarct volumes were quantified by TTC staining 3 d after tMCAO. *N* = 5–6/group. (**J**) Aged male mice were subjected to dMCAO and treated with vehicle or TD139 (0.4 mg/kg, starting 2 h after tMCAO, then daily for 2 d). Infarct volumes were quantified by MAP2 staining 3 d after dMCAO. *N* = 7–9/group. Neuron intersection data in **C** and **G** are plotted as mean ± SEM. Other data are plotted as mean ± SD. *P < 0.05, **P < 0.01, ***P < 0.001. Two-tailed, unpaired Student’s *t* test (**F**, **H**, and **J**) or 1-way (**B**, **D**, and **I**) or 2-way (**C** and **G**) ANOVA and Bonferroni’s test.

We then tested in vitro whether inhibiting GAL3 activity in MDMø can provide neuronal protection using BMDM-neuron cocultures. Neurons were cultured in the bottom chamber of a transwell system and subjected to 60 min oxygen glucose deprivation (OGD) to induce ischemic neuronal damage, which was quantified by reduced coverage of microtubule-associated protein 2–positive (MAP2^+^) neurons and fewer neuron numbers (Supplemental Figure 4G). Sholl analyses further revealed shortened mean primary branch lengths (Supplemental Figure 4H). The WT or GAL3-KO BMDMs were cultured in transwell inserts and exposed to brain lysates for 6 h. Untreated or lysate-activated BMDMs were then washed, added on top of the bottom chamber housing OGD neurons, and cocultured with OGD neurons for 18 h (Figure 5A). Neurons cocultured with lysate-activated WT BMDMs, but not GAL3-KO BMDMs, showed smaller MAP2^+^ staining areas (Figure 5B). Sholl intersection profiles demonstrated less neuronal arborization, including fewer branches at a given distance from the soma (Figure 5C) and reduced mean primary branch lengths (Figure 5D) in neurons cocultured with lysate-activated WT BMDMs, suggesting that the expression of GAL3 in BMDMs rapidly induces neurotoxicity.

Next, we inhibited GAL3 activity using the selective, cell-permeable inhibitor TD139 in WT BMDMs during the period of exposure to brain lysates (Figure 5E). TD139 reduced the neurotoxicity of lysate-activated BMDMs, as shown by enlarged areas of MAP2^+^ neuronal staining (Figure 5F), more intersections at proximal distances (Figure 5G), and elongated mean primary branch lengths (Figure 5H) compared with the vehicle control.

### Pharmacologic inhibition of GAL3 diminishes acute brain infarct early after stroke.

Next, we tested the therapeutic effects of TD139 in young male mice subjected to the 60 min tMCAO model. TD139 (0, 0.2, 0.4, 0.8, and 1.6 mg/kg body weight) or an equal volume of vehicle was administered intravenously, initially at 2 h after surgery and then daily for 2 d. Blinded outcome assessments showed that 0.4, 0.8, and 1.6 mg/kg TD139 reduced brain infarction 3 d after tMCAO (Figure 5I). The lowest dose that achieved the best protection, 0.4 mg/kg, was employed for subsequent assessments. We also tested the therapeutic effects of TD139 in 20-month-old mice using a less severe distal MCAO (dMCAO) model. TD139 treatment (0.4 mg/kg at 2 h, 1 d, and 2 d after dMCAO) also reduced brain infarct 3 d after dMCAO in aged mice (Figure 5J).

### High GAL3 expression in MDMø amplifies cathepsin induction and leakage after stroke, thereby enlarging brain lesions.

**Figure 6:**
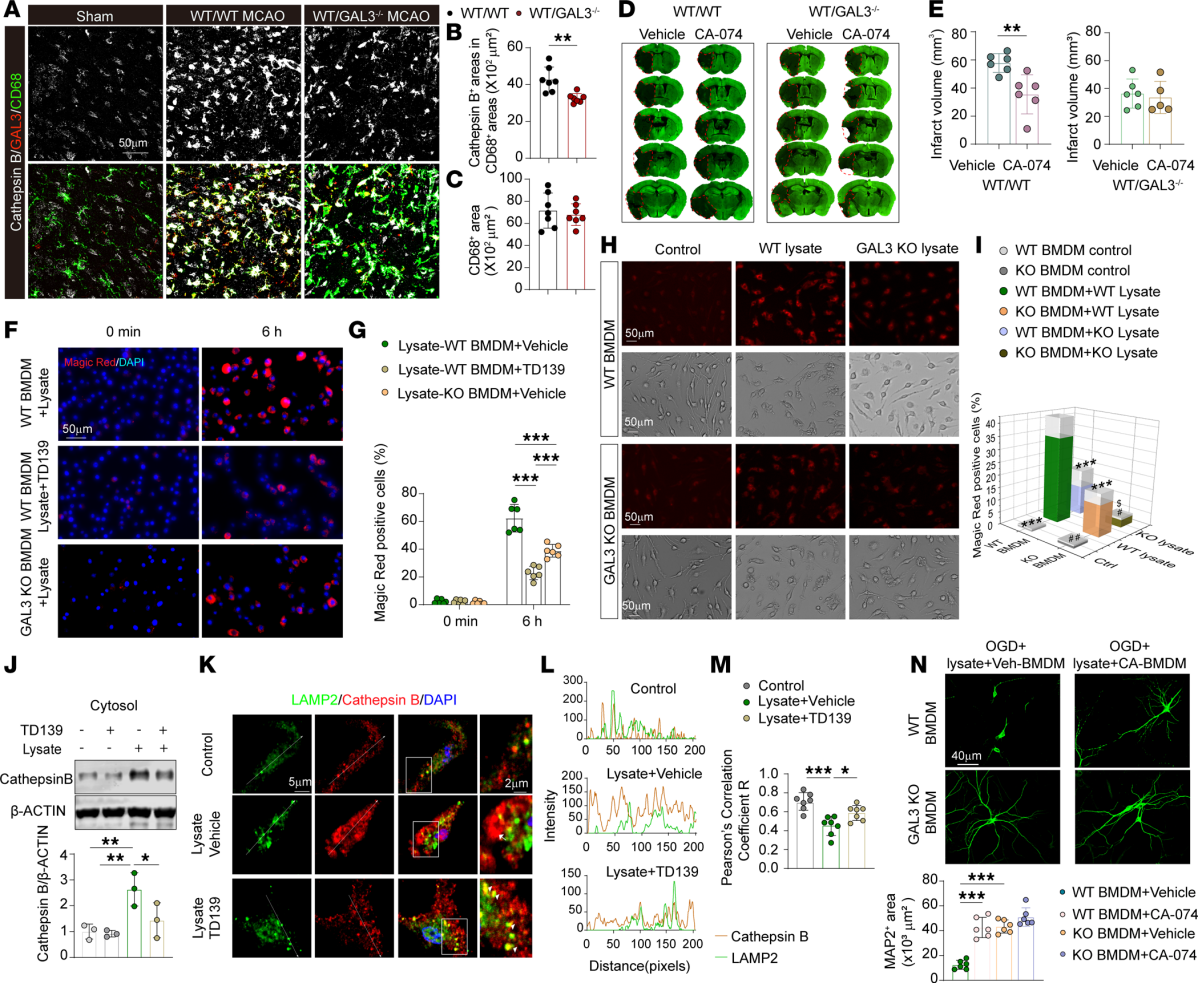
High GAL3 expression in MDMø amplifies cathepsin induction and leakage after stroke, thereby enlarging brain lesions. (**A**) Representative immunostaining of cathepsin B/GAL3/CD68 in WT/WT and WT/GAL3^–/–^ chimera mice 3 d after tMCAO. Scale bar: 50 μm. (**B**) Quantification of cathepsin B^+^ staining areas within CD68^+^ areas of macrophages. (**C**) Quantification of CD68^+^ areas. *N* = 7/group. (**D**) MAP2 staining of brain in vehicle- or CA-074–treated (10 mg/kg, i.v., 2 h after tMCAO and then daily for 2 d) WT/WT or WT/GAL3^–/–^ chimeric mice 3 d after tMCAO. (**E**) Quantification of brain infarct volume. *N* = 5–6/group. (**F**) Cathepsin B activity was analyzed using the Magic Red cathepsin kit in GAL3-KO or WT BMDMs with vehicle or TD139 (10 μM) treatment. Scale bar: 50 μm. (**G**) Quantification of the percentages of Magic Red^+^ cells among total cells. *N* = 6/condition. (**H**) Representative Magic Red staining in WT and GAL3-KO BMDMs treated with or without WT or GAL3-KO lysate for 6 h. Scale bars: 50 μm. (**I**) Quantification of the percentages of Magic Red^+^ cells among total cells. *N* = 6/condition. Gray blocks show SD. ***P < 0.001 versus (WT BMDM + WT lysate); ^#^*P* < 0.05, ^##^*P* < 0.01 versus (KO BMDM + WT lysate); ^$^*P* < 0.05 versus (WT BMDM + KO lysate). (**J**) Immunoblot and quantification of cathepsin B expression in cytosol of BMDMs under the indicated conditions for 6 h. *N* = 3/condition. (**K**) Coimmunostaining of LAMP2 and cathepsin B in BMDMs under specified conditions for 6 h. Scale bars: 5 μm, 2 μm (zoom). (**L**) Fluorescence intensity profiles of cathepsin B and LAMP2. (**M**) Pearson’s correlation analyses of LAMP2 and cathepsin B. *N* = 7/condition. (**N**) BMDMs in inserts treated with brain lysates and CA-074 (10 μM) or vehicle were cocultured with OGD neurons. Coverage areas of MAP2-stained neurons were quantified. *N* = 6/condition. Scale bar: 40 μm. *P < 0.05, **P < 0.01, ***P < 0.001. Two-tailed, unpaired Student’s *t* test (**B**, **C**, and **E**) or 1-way (**G**, **J**, **M**, and **N**) or 2-way (**I**) ANOVA and Bonferroni’s test.

Lysosomes house an acidic lumen containing hydrolytic enzymes such as cathepsins. We found an increased area of cathepsin B expression in CD68^+^ phagocytes in the ischemic brain region in WT/WT mice compared with WT/GAL3-KO chimera mice 3 d after tMCAO (Figure 6, A and B), suggesting that lysosomal accumulation of GAL3 is accompanied by the induction of lysosomal content. No difference was observed in the area of CD68^+^ cells between the 2 types of chimera mice (Figure 6C). Lysosomal leakage of lumen contents provokes inflammatory cascades and cell damage under pathological conditions (26). To test the importance of lysosomal content in GAL3-mediated detrimental MDMø responses and acute ischemic brain injury, we injected mice 2 h after tMCAO with an inhibitor of cathepsin B, CA-074-Me (CA-074). CA-074 treatment significantly reduced ischemic brain injury in WT/WT mice but failed to provide additional protection in WT/GAL3-KO chimera mice (Figure 6, D and E), confirming that GAL3 inhibition may protect the brain by ameliorating the detrimental effects of cathepsin B.

Next, we exposed BMDMs to WT brain lysates, in the presence or absence of TD139. Cathepsin activity, as measured by the Magic Red cathepsin assay, was induced in WT BMDMs at different time points after exposure to WT brain lysates, and this effect was ameliorated by TD139 or GAL3-KO in BMDMs (Figure 6, F–I, and Supplemental Figure 4, I and J). Brain lysates from GAL3-KO mice, which are devoid of exogenous GAL3, also induced cathepsin activity at a lower level in WT BMDMs but failed to induce cathepsin in GAL3-KO BMDMs (Figure 6, H and I). These data suggest that both exogenous GAL3 and GAL3 synthesized in BMDMs contribute to cathepsin induction from stimulated BMDMs.

Western blotting confirmed that the exposure to brain lysates increased cathepsin B levels in the BMDM cytosolic fraction (i.e., without lysosomes), and this effect was inhibited by TD139 treatment (Figure 6J). The expression of cathepsin B was also visualized in relation to lysosomes by immunofluorescent staining (Figure 6K). Cathepsin B expression was highly correlated with LAMP2 signal in untreated control BMDMs. Treatment with brain lysates raised cathepsin B expression and induced the spatial dissociation of cathepsin B from LAMP2 (Figure 6, L and M). TD139 treatment reduced cathepsin B levels and reduced the spatial dissociation of cathepsin B from LAMP2. These data suggest that GAL3 is important for induction of cathepsin expression as well as cytosolic escape of cathepsin from the MDMø lysosome upon noxious stimulations.

We then measured lysosomal membrane permeability by assessing the ability of lysosomes to retain fluorophore-conjugated dextran. WT BMDMs were preloaded with 10 kDa Alexa Fluor 555–dextran and 40 kDa fluorescein-dextran, which accumulates within punctuate structures inside lysosomes in unstimulated cells (Supplemental Figure 4K). Cells loaded with dextran were then exposed to brain lysates in the presence or absence of TD139. Brain lysate exposure caused a release of 10 and 40 kDa dextran from the lysosomes, resulting in diffuse cytosolic distribution patterns and lower fluorescence intensities 6 h after exposure (Supplemental Figure 4, K and L). TD139 treatment reduced the leakage of lysosomal dextran into the cytosol, as manifested by reduced diffusion (Supplemental Figure 4K) and increased intensity (Supplemental Figure 4L) of both small and large size dextran molecules. We also measured lysosomal membrane permeability after brain lysate exposure using acridine orange (AO), which accumulates in acidic lysosomes and emits red fluorescence at low pH (Supplemental Figure 4M). AO emits green fluorescence in the neutral pH environment in the cytosol (27, 28). We observed that AO-labeled red puncta increased significantly in TD139-treated BMDMs compared with vehicle-treated BMDMs 6 h after brain lysate exposure (Supplemental Figure 4N), suggesting reduced lysosomal leakage after TD139 treatment.

We then treated WT BMDMs with the cathepsin B inhibitor CA-074 or vehicle during brain lysate exposure. CA-074+lysate-treated BMDMs enhanced the survival of OGD neurons compared with vehicle+lysate-treated BMDMs, as shown by increased areas of MAP2^+^ neuronal staining in BMDM-neuron cocultures within the transwell system (Figure 6N). GAL3-KO BMDMs showed larger areas of MAP2^+^ neuronal staining, and exposure of GAL3-KO BMDMs to CA-074 treatment did not further enhance the survival of OGD neurons (Figure 6N).

### GAL3-mediated cathepsin induction enhances NLRP3 expression in MDMø and amplifies post-stroke neuroinflammation.

**Figure 7:**
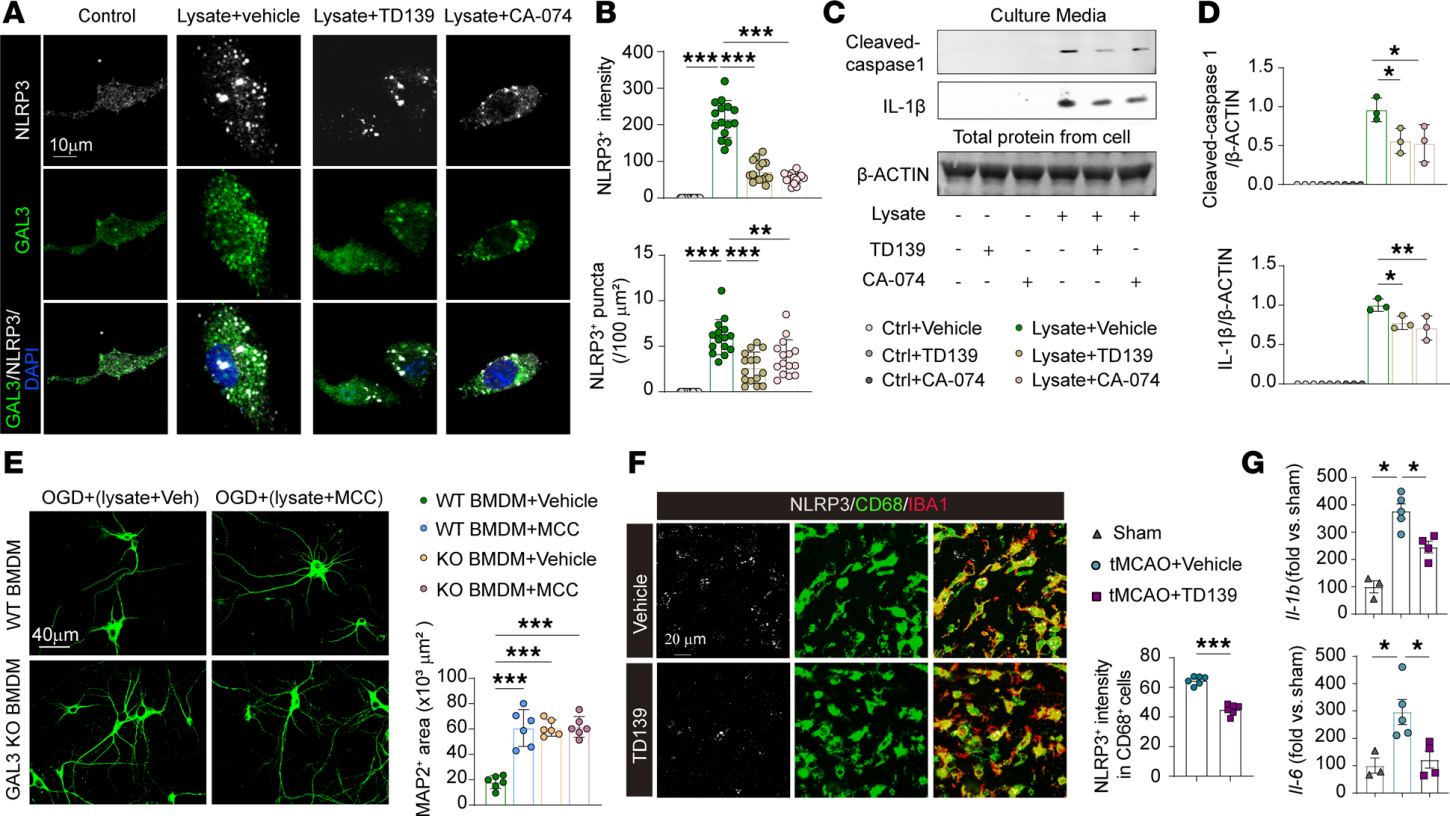
GAL3-mediated cathepsin induction enhances NLRP3 expression in MDMø and amplifies post-stroke inflammation in the CNS. (**A**) Immunostaining of NLRP3 and GAL3 in cultured BMDMs under specified conditions for 6 h. Scale bar: 10 μm. (**B**) Quantification of NLRP3 intensity and number of NLRP3^+^ puncta per 100 μm^2^ cell area within BMDMs in indicated groups. *N* = 15 randomly selected BMDMs from 3 independent experiments. (**C**) Immunoblot of cleaved caspase-1 and active IL-1β in culture media of BMDMs under specified conditions for 6 h. β-ACTIN from corresponding cells was used as control. (**D**) Quantification of cleaved caspase-1 and IL-1β in indicated groups. *N* = 3/condition. (**E**) BMDMs in cell culture inserts were treated with brain lysates and MCC950 (10 μM) or vehicle controls for 6 h. The treated BMDMs were then cocultured with OGD neurons for 18 h. Representative images show MAP2 immunostaining under indicated conditions. Coverage areas of MAP2-stained neurons were quantified. *N* = 6 samples per condition. Scale bar: 40 μm. (**F**) Immunostaining of NLRP3, CD68, and IBA1 3 d after tMCAO in WT mice treated with vehicle or TD139. NLRP3 intensity within CD68^+^ cells was quantified. *N* = 6/condition. Scale bar: 20 μm. (**G**) RNA levels of *Il-1b* and *Il-6* expression in ipsilateral brain lysates collected 5 d after tMCAO. *N* = 3–5/group. *P < 0.05, **P < 0.01, ***P < 0.001. Two-tailed, unpaired Student’s *t* test (**F**) or 1-way ANOVA and Bonferroni’s test (**B**, **D**, **E**, and **G**).

Lysosomal disruption is known to activate the NLRP3 inflammasome in macrophages by releasing cathepsins (29, 30). We therefore evaluated the effects of GAL3 on inflammasome activation in BMDMs after brain lysate exposures. NLRP3 staining intensities and the numbers of NLRP3^+^ puncta/100 μm^2^ (Figure 7, A and B) increased upon brain lysate exposure. Both TD139 and CA-074 lowered this rise in NLRP3 expression (Figure 7, A and B). Western blotting also showed elevated secretion of cleaved caspase-1 and IL-1β in brain lysate–treated BMDMs, and these effects were similarly inhibited by TD139 or CA-074 (Figure 7, C and D).

When WT BMDMs were treated with the NLRP3 inhibitor MCC during brain lysate exposure and then added on top of OGD neurons, MCC reduced the neurotoxicity of activated WT BMDMs, as shown by increased areas of MAP2^+^ neuronal staining (Figure 7E). Applying MCC to GAL3-KO BMDMs, however, showed no additional effects on neuron survival (Figure 7E).

Consistent with our in vitro observations, we found decreased NLRP3 expression in CD68^+^ cells and fewer NLRP3^+^CD68^+^ cells in the ischemic area in WT/GAL3-KO mice compared with WT/WT mice 3 d after tMCAO (Supplemental Figure 5A). CA-074 treatment reduced the number of NLRP3^+^CD68^+^ cells in the ischemic brain, whereas no differences in the total number of CD68^+^ cells were observed (Supplemental Figure 5B). Similarly, TD139 treatment decreased NLRP3 expression in CD68^+^ cells (Figure 7F) and reduced cerebral upregulation of inflammatory cytokines IL-1β and IL-6 in the lesioned hemisphere (Figure 7G). In addition, flow cytometry analyses confirmed that the NLRP3 expression level in blood monocytes was reduced in TD139-treated aged mice (Supplemental Figure 5, C and E).

### Transient GAL3 inhibition early after stroke improves long-term outcomes.

**Figure 8:**
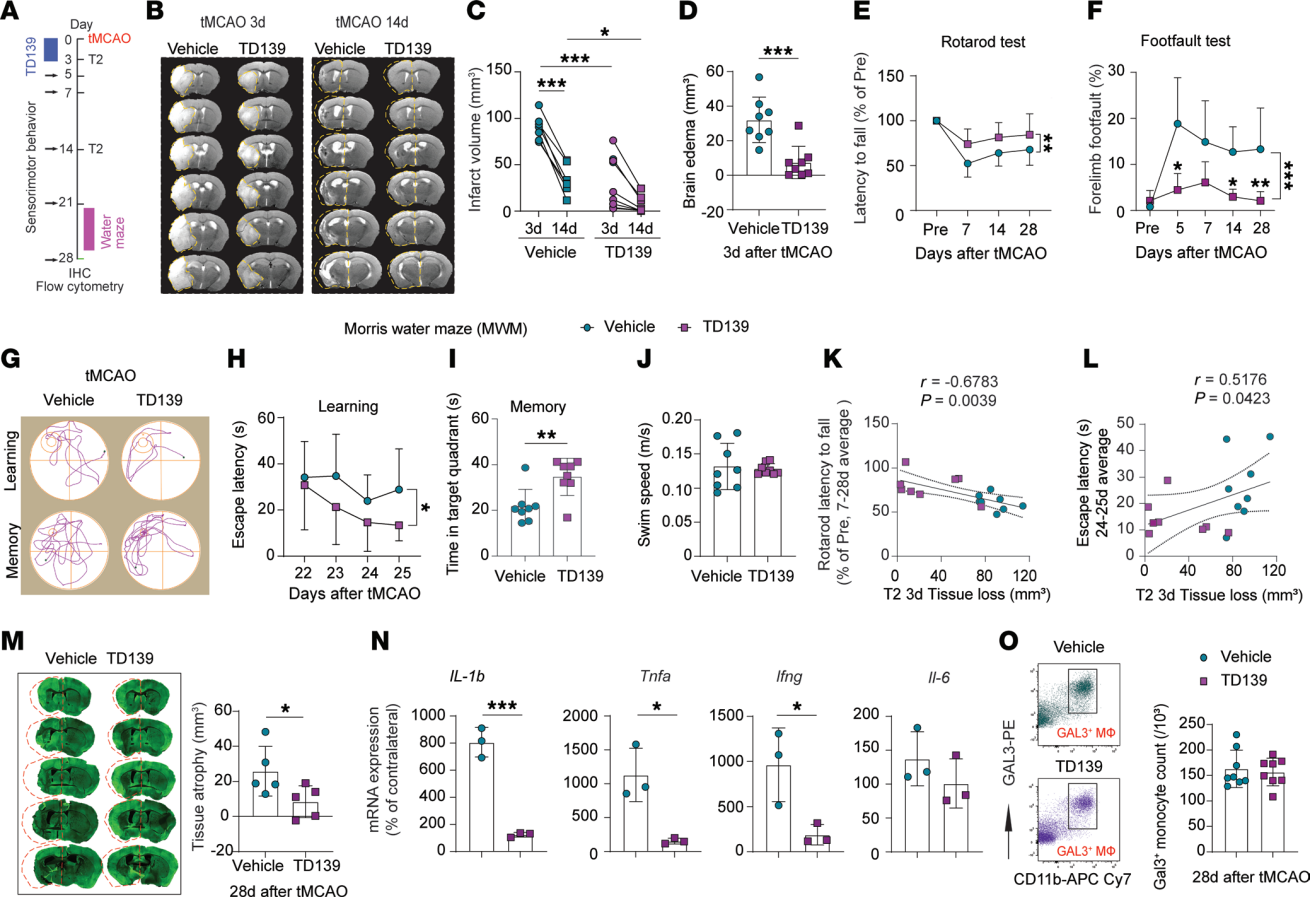
Transient GAL3 inhibition improves long-term stroke outcomes. (**A**) Experimental design. (**B**) Representative images of T2 MRI scans. For tMCAO 3d, the yellow dashed lines trace the border of infarct lesion. For tMCAO 14d, the areas of contralesional hemisphere are reflected on the ipsilesional hemisphere (yellow dashed lines). (**C** and **D**) Quantification of T2 signals revealed brain infarcts (**C**) and brain edema (**D**). *N* = 8/group. (**E** and **F**) Motor abilities were assessed using rotarod (**E**) and foot fault (**F**) tests. *N* = 8/group. (**G**–**J**) Cognitive performance was assessed using the Morris water maze. *N* = 8/group. (**G**) Representative swimming trace during learning and memory tests. Small circles show the position of the platform. (**H**) The latencies of finding the escape platform during the learning phase. (**I**) Time spent in the target quadrant where platform was previously located during the learning phase. (**J**) Average swimming speed. (**K**) Analysis of Pearson’s correlation between the average latency to fall on the rotarod test (days 7–28) and tissue loss measured 3 d after MCAO. (**L**) Analysis of Pearson’s correlation between the average escape latency in the water maze test (days 24–25) and tissue loss measured 3 d after MCAO. (**M**) MAP2 staining and quantification of brain atrophy 28 d after tMCAO. The areas of contralesional hemisphere are reflected on the ipsilesional hemisphere (red dashed lines). *N* = 5/group. (**N**) PCR analysis of mRNA expression of *Il-1b*, *Tnfa*, *Ifng*, and *Il-6*. Data are expressed as percentages of contralateral levels. *N* = 3/group. (**O**) Flow cytometry detected the number of GAL3^+^CD11b^+^ monocytes 28 d after tMCAO in the blood of young WT mice treated with vehicle or TD139. *N* = 8/group. *P < 0.05, **P < 0.01, ***P < 0.001. Two-tailed, unpaired Student’s *t* test (**D**, **I**, **J**, **M**, **N**, and **O**), 2-way ANOVA (**C**), or 2-way repeated measures ANOVA (**E**, **F**, and **H**) and Bonferroni’s test.

We tested if TD139-afforded brain protection can be sustained into the chronic injury phase. Young male C57BL/6 mice (5 months old) were subjected to 60 min tMCAO and randomly assigned to receive TD139 (i.v., 0.4 mg/kg BW) or an equivalent volume of vehicle at 2 h after tMCAO and then daily for 3 d (Figure 8A). T2 scans were collected 3 and 14 d after tMCAO (Figure 8B). TD139 treatment in the early phases of stroke mitigated brain tissue loss (Figure 8C) and brain edema (Figure 8D) 3 d after tMCAO. This pattern lasted at least 14 d after tMCAO (Figure 8C). TD139 treatment also improved sensorimotor performance in the rotarod (Figure 8E) and foot fault (Figure 8F) tests. The Morris water maze test was performed 22–26 d after tMCAO (Figure 8, G–J). TD139-treated mice exhibited better learning capacity, as shown by reduced latencies to find the hidden platform (Figure 8H), and superior memory, as shown by increased times spent in target quadrant when the platform was removed (Figure 8I), compared with vehicle-treated mice. There was no change in swim speeds (Figure 8J), revealing that the protective effects of TD139 were not attributed to differences in gross locomotor abilities. A negative correlation was observed between tissue loss in T2 and the latency to fall in the rotarod test (Figure 8K), while a positive correlation was noted between tissue loss and average escape latencies on days 24 and 25 of the water maze (Figure 8L). Consistent with improved behavior performance, TD139-treated mice exhibited less brain tissue loss 28 d after tMCAO (Figure 8M). TD139-treated mice also displayed lower cerebral expression of inflammatory cytokines *Il-1b*, *Tnfa*, and *Ifng* compared with vehicle-treated mice, with minimal effects on *Il-6* expression 28 d after tMCAO (Figure 8N). Flow cytometry analyses confirmed that short-term TD139 treatment early after stroke had no long-term effect on the number of circulating GAL3^+^ monocyte/Mø 28 d after tMCAO (Figure 8O).

## Discussion

The present study dissects the temporally specific roles of GAL3 in MDMø in the context of ischemic brain injury. Our evaluation of mice with global KO of GAL3 reveals critical biphasic roles after ischemic stroke. GAL3 KO decreased brain lesion sizes at 3 and 7 d after stroke and improved sensorimotor functions within 1 week after stroke, suggesting an early destructive role of this molecule. However, the early neuroprotection in GAL3-KO mice waned over the course of 4 weeks. The sensorimotor functions improved over time in WT mice but not GAL3-KO mice, canceling differences between the 2 genotypes about 7 d after stroke. Long-term cognitive functions deteriorated in GAL3-KO mice. These data suggest that GAL3 plays dynamic roles, enhancing stroke recovery in the chronic injury phase, in line with previous studies demonstrating a role for GAL3 in post-stroke angiogenesis and neurogenesis (10, 16). Although we observed reduced brain injury 3 d after tMCAO, it is important to note that Lalancette-Hebert et al. reported opposing results (14). Indeed, the current literature suggests that GAL3 may exert both neurodestructive (11–13) and neuroprotective (14–16) roles early after stroke. Although the conclusions from different studies cannot be easily reconciled due to the differences in age/sex of animals, stroke models, and timing of outcome measurements, these studies clearly reflect our lack of understanding of the diverse functions of GAL3 and highlight the importance of disentangling the cell- and phase-specific functions of this molecule.

Analyses of several recent scRNA-seq datasets (3, 19) consistently show far higher expression of *Lgals3* in infiltrating MDMø after experimental stroke compared with other myeloid cells. This is in line with reports showing GAL3 upregulation in monocytes/macrophages in response to different inflammatory cytokines, including GM-CSF (31), TNF-α, and IFN-γ (32), all of which are increased in the ischemic brain or cell debris. Our immunostaining and flow cytometry results further confirmed that GAL3 expression in infiltrating MDMø was much higher than in microglia 3–5 d after tMCAO, prompting us to explore the importance of MDMø in the destructive roles of GAL3 at the early phase of stroke. We provided multiple lines of evidence (using chimeric WT mice reconstituted with GAL3-KO BM, WT mice whose monocytes were replaced by GAL3-KO monocytes, and Mø-specific GAL3-KO mice) to demonstrate that GAL3 expression in MDMø exacerbated acute ischemic brain injury. The use of BMDM-neuron cocultures strengthened our interpretations of the in vivo observations by confirming that exposure to brain lysates induced GAL3 expression in BMDMs to mediate a neurotoxic response. Consistent with our results, previous studies showed that increased GAL3 expression in inflammatory macrophages enhances smooth muscle cell apoptosis (33). In addition, infiltration of phagocytic GAL3-expressing macrophages into the diabetic brain promotes vessel loss after brain injury (34). Conversely, others have reported that GAL3 expression in M2-like macrophages protects against acute insults in control and fibrotic mice by inhibiting pyroptosis (35) and reduces neural cell death upon parasitic infections by promoting efferocytosis and resolution of inflammation (36). The opposing conclusions are likely to be attributed to the pleiotropic functions and disparate subcellular localizations of GAL3, as well as activation of distinct signaling mechanisms by GAL3, collectively leading to tissue- and disease-specific as well as potentially spatiotemporally dependent reactions of macrophages (37).

Although this study focuses on the impact of GAL3 on MDMø, it should be noted that cellular expression of GAL3 after stroke also encompasses microglia, border-associated Mø, DCs, and peripheral neutrophils at lower levels. Using monocyte/Mø depletion and replacement strategies, we confirmed the detrimental impact of GAL3^+^ MDMø on brain infarct, but we did not exclude the influence of GAL3 in other myeloid cells upon stroke outcomes. For example, GAL3 can act as an endogenous ligand for TLR4 and TREM2 receptors, which are known to sustain microglial activation and prolong inflammatory response in stroke (11) and Alzheimer’s disease (38), respectively. GAL3 has also been reported to promote the recruitment and activation of neutrophils during bacterial infection (39) and enhance neutrophil apoptosis and efferocytosis during inflammation resolution (40). However, we observed GAL3 expression only in circulating neutrophils but not in brain infiltrating neutrophils. To our knowledge, there is no direct evidence of the impact of neutrophil GAL3 on stroke outcomes. Future studies using microglia or neutrophil-specific GAL3-KO mice and primary cell cultures would help elucidate the diverse cell-specific functions of GAL3 in the acute phase of stroke injury.

In the context of acute ischemic brain injury, we discovered a mechanism whereby GAL3 mediates a neurotoxic response in MDMø through enhancement of lysosomal-related inflammation. Cell demise after stroke generates debris, which requires prompt lysosomal digestion to prevent secondary inflammatory injury and clear a path for regenerative processes. MDMø are professional scavengers of debris and entrap toxic cellular fragments in phagosomes (6). The lysosome is a digestive apparatus that degrades engulfed materials after fusing with phagosomes into an autophagolysosome. However, lysosomal membrane permeabilization after phagocytosis results in leakage of intracellular contents, such as cathepsins, which provoke oxidative stress, inflammatory cascades, and cell damage (26). We demonstrate that upregulation of GAL3 in MDMø was colocalized with lysosomal markers upon exposure to brain lysates or after infiltration into the ischemic brain. As a carbohydrate-binding protein, GAL3 can bind to the glycans that are normally present in the lysosomal lumen but are only exposed to the cytosol upon lysosomal disruption, resulting in the accumulation of GAL3 in damaged lysosomes and formation of GAL3^+^ puncta (41, 42). We found that pharmacologic GAL3 inhibition or genetic deletion from MDMø resulted in physical dissociation of GAL3 from lysosomes, an effect that was accompanied by lower expression of cathepsin, reduced escape of cathepsin from the lysosomal lumen, and decreased lysosomal leakage. In contrast to the current observation that GAL3 enhances lysosomal leakage, others reported that GAL3 coordinates a reparative cellular response to lysosomal damage and promotes lysosomal repair and functional restoration (41). In the latter study, transformed HeLa cells were treated with a lysosomal membrane rupturing agent, whereas our in vitro experiments employed cultured macrophages challenged with brain lysates. It is conceivable that the phagocytic activities of macrophages exposed to high amounts of damage-associated molecular patterns from cellular debris in brain lysates dramatically enhance lysosomal activation and accumulation, which may differ in significant ways from the lysosomal response in damaged HeLa cells. Accordingly, the destructive roles of GAL3 in lysosomal integrity are consistent with reports in other disease models. For example, pathological increases in GAL3 caused the accumulation of ruptured lysosomes in microglia and enhanced neuroinflammation in experimental Huntington’s disease (23).

Lysosomal disruptions may activate the NLRP3 inflammasome in Mø by the release of cathepsins (29, 30). Cathepsin B has been reported to increase in infiltrating MDMø and microglia after neonatal hypoxia/ischemic injury and is partly responsible for the ensuing damage (43). The mechanistic implication that cathepsin B mediates GAL3-induced neurotoxic MDMø is supported by the neuroprotective impact of cathepsin B inhibition in WT mice or WT MDMø, an effect that mimicked the deletion of GAL3. In line with previous reports that cathepsin B triggers NLRP3 inflammasome activation when released into the cytosol under pathological conditions (29, 30), we have shown that GAL3 or cathepsin B inhibition reduced NLRP3 expression, inflammasome activation, and inflammatory cytokine production in MDMø. Collectively, our data support a role for GAL3 in lysosomal release of cathepsin and subsequent activation of NLRP3 inflammasome-dependent inflammatory reactions in activated MDMø during the early phase after stroke, thereby contributing significantly to ischemic brain injury. The enhanced inflammasome signaling and inflammation may upregulate the expression and function of additional GAL3, forming a self-amplifying cascade that exacerbates brain injury. It is possible that other mechanisms also contribute to GAL3-mediated neurotoxicity in BMDMs. For instance, GAL3-dependent TLR4 activation has been documented to sustain microglial activation, prolonging the inflammatory response under ischemic stroke conditions (11). Additionally, GAL3 and NFκB can be regulated in a positive feed-forward loop in microglia to enhance inflammation (23). Whether these mechanisms contribute to GAL3-mediated inflammation in MDMø warrants further investigation.

One question raised by our observations is the mechanism whereby GAL3 enhances lysosomal leakage. A recent study showed that high expression of GAL3 impedes clearance of damaged lysosomes by inhibiting lysophagy (23). As a result, ruptured lysosomes continue to accumulate in phagocytes and spill their contents. An alternative explanation for increased lysosomal leakage in GAL3^+^ MDMø might be related to their high phagocytic activity. Sano et al. demonstrated that intracellular GAL3 in macrophages accelerates the phagocytic clearance of erythrocytes (44). Another study reported that extracellular GAL3 augments phagocytosis of apoptotic neutrophils by functioning as an opsonin (45). An abrupt increase in phagocytosis may lead to dramatic lysosomal reorganization and rapid expansion of lysosomal volume (46). Large lysosomes are more susceptible to disruption (47). Moreover, some of the phagocytosed cargo that ends up in autophagolysosomes may rupture lysosomal membranes (48). Therefore, rapid and aggressive phagocytic activity may be induced by GAL3 in macrophages via both intracellular and extracellular pathways, perhaps explaining why GAL3 elicited lysosomal leakage. In line with this possibility, we found that GAL3 expression increased lysosomal diameter and area in activated BMDMs, which may increase the probability of lysosomal disruption. Our data also showed that GAL3 in both brain lysates and BMDMs contributed to cathepsin activation in BMDMs, in line with the contribution of both extracellular and intracellular GAL3 to phagocytosis (44, 45). Future studies on the effects of GAL3 on phagocytosis and lysophagy in MDMø after stroke would provide additional insight into the mechanisms by which GAL3 modulates lysosomal integrity.

We found that transiently inhibited GAL3 with TD139 at the early phase reduced acute ischemic brain lesions. The relatively short plasma half-life of TD139 (25) helps to ensure that the acute treatment regimen does not disturb long-term proregenerative functions of GAL3. Therefore, TD139-afforded early neuroprotection culminates in improvements in sensorimotor and cognitive functions, as well as reduction of brain atrophy at the late phase of stroke injury. TD139 is approved as an investigational new drug for idiopathic pulmonary fibrosis (IPF). Our dose escalation showed that 0.4 mg/kg TD139 was optimal and efficacious in both young and aged mice. The 0.4 mg/kg dose has been calculated as equivalent to approximately 1.2 mg/m^2^ in humans (49). Inhaled TD139 reduces GAL3 expression in alveolar macrophages for the treatment of human IPF at 3 mg/day (~1.85 mg/m^2^/day) and 10 mg/day (25), doses that are higher than the effective dose in our stroke model. This dose range of TD139 is safe and well tolerated in healthy subjects and IPF patients according to randomized, double-blind, multicenter, and placebo-controlled phase I/IIa studies (25). Therefore, early GAL3 inhibition by transient peripheral application of a safe dose of TD139 may have translational potential in the stroke clinic. Further studies are warranted to test if TD139 treatment can be initiated at later time points after stroke, to increase the feasibility of drug application in the clinic.

In summary, our study shows that high expression of GAL3 in MDMø induces cathepsin B expression in lysosomes and the escape of cathepsin B into the cytoplasm, triggering inflammasome activation and exacerbating cerebral infarcts. Thus, inhibition of GAL3 by TD139 in the acute phase of stroke injury may serve as a therapeutic strategy to divert MDMø from a destructive phenotype while preserving their reparative functions.

## Methods

Additional methods information can be found in the supplemental materials.

### Sex as a biological variable.

Our study exclusively examined male mice because young female mice have been shown to be protective against ischemic stroke (50). Sexual dimorphism in GAL3 has also been reported (51). Further studies would be necessary to elucidate if and how sex modifies the association of GAL3 with stroke outcomes.

### Animals.

C57BL/6, GAL3-KO (GAL3^–/–^), Ai14 Rosa26^RFP^, Rag1-KO, and *LysM*^Cre+/–^ mice were obtained from The Jackson Laboratories. The *Lgals3*^fl/fl^ mouse line was generated in-house. The *Ccr2*^CreER+/–^ mouse line was a gift from Chia-Yi Kuan, University of Virginia, Charlottesville, Virginia, USA. The myeloid cell–specific GAL3-KO (*LysM*^Cre+/–^*Lgals3*^fl^/^fl^) mice were bred from *Lgals3*^fl/fl^ and *LysM*^Cre+/–^ mice. The *Ccr2*^CreER+/–^Ai14-RFP mice were bred from Ai14 Rosa26^RFP^ mice and *Ccr2*^CreER+/–^ mice. The expression of RFP in macrophages was induced in *Ccr2*^CreER+/–^Ai14-RFP mice by intraperitoneal injection of 4-hydroxytamoxifen (0.1 mg in 100 μL corn oil, daily for 5 days). All efforts were made to minimize animal suffering and the number of animals used. All animals were housed in a temperature- and humidity-controlled facility with a 12 h light/dark cycle. Food and water were available ad libitum. All procedures were conducted by investigators blinded to genotype and experimental group assignments.

### Reagents.

Stock solutions of TD139, a specific GAL3 inhibitor (TargetMol) and CA-074 methyl ester, a specific cathepsin B inhibitor (MedChem Express) were prepared at concentrations of 10 mM for in vitro use and 10 mg/mL for in vivo use. Both reagents were dissolved in DMSO. For in vitro experiments, stocks were diluted in media to a final concentration of 10 μM; for in vivo experiments, the final doses were 0.4 mg/kg BW (optimal dose) for TD139 and 10 mg/kg BW for CA-074.

### Antibodies.

The antibodies used were as follows. For immunostaining, we used goat anti-IBA1 (Abcam, ab5076), rabbit anti-NLRP3 (Abcam, ab263899), rabbit anti-CD11c (Abcam, ab219799), rat anti-GFAP (Invitrogen, 13-0300), rat anti-CD68 (Invitrogen, 14-0681-82), goat anti-CD31 (R&D Systems, AF3628), goat anti-MPO (R&D Systems, AF3667), mouse anti-GAL3 (Santa Cruz Biotechnology, sc-32790), rabbit anti-NEUN (Sigma-Aldrich, ABN78), rat anti-CD206 (Bio-Rad, MCA2235), and rabbit anti-MAP2 (Sigma-Aldrich, AB5622). For flow cytometry, we used CD11b-BUV737 (BD Biosciences, 612800), LY6G-BUV395 (BD Biosciences, 563978), CXCR4 BV510 (BD Biosciences, 563468), CD8 BV510 (BD Biosciences, 563068), CD11b-APC-Cy7 (BD Biosciences, 557657), CD11c BV421 (BD Biosciences, 562782), F4/80-BV605 (BD Biosciences, 743281), CD4 Pacific blue/eFluor 450 (Invitrogen, 48004182), CD11b-APC-eFluor 780 (Invitrogen, 47011282), CD45 PerCP-Cy5.5 (Invitrogen, 45045182), GAL3-PE (Invitrogen, 12530182), CD19 BV650 (BioLegend, 115541), NK1.1 BV785 (BioLegend, 108749), and NLRP3 APC (R&D Systems, IC7578A). For Western blotting, we used rabbit anti–cleaved caspase-1 (Cell Signaling Technology, 89332S), mouse anti-GAL3 (Santa Cruz Biotechnology, sc-32790), mouse anti–IL-1β (R&D Systems, AF-401-NA), rabbit anti–cathepsin B (Abcam, ab214428), and mouse anti–β-actin (R&D Systems, MAB8929).

### tMCAO.

tMCAO was induced in young (5-month-old) and aged (20-month-old) male mice using the intraluminal occlusion method to block the left middle cerebral artery (MCA) for 60 min as we previously described (21). Regional cerebral blood flow (rCBF) was monitored using a 2D laser speckle imaging system (PeriCam PSI System) to confirm successful occlusion. Mice showing a rCBF reduction of >70% from pre-MCAO baseline levels were included in subsequent analyses. Sham-operated mice underwent identical surgical procedures, except for MCAO. Body temperature was maintained at 37°C ± 0.5°C throughout the surgery with a temperature-regulated heating pad.

### Permanent dMCAO.

Permanent dMCAO was induced in 20-month-old male mice as we previously described (52). rCBF was measured using laser Doppler flowmetry. Mice showing an rCBF reduction of >70% from pre-MCAO baseline levels were included in the study. Sham-operated animals underwent identical anesthesia and surgical procedures but were not subjected to dMCAO.

### BM isolation and differentiation of BMDMs.

BM was isolated from the femur and tibia of WT or GAL3-KO male mice at 8–10 weeks of age as we previously described (21). BM cells were then cultured in plastic dishes (100 mm diameter) in macrophage culture medium (RPMI-1640, 10% FBS, 20% L929 conditioned medium, and 1% penicillin-streptomycin) to promote the growth of BMDMs. After 7 days in culture, the media was supplemented with 20 ng/mL macrophage CSF and cultured for 3 more days before further experiments.

### Irradiation and BM transplantation.

To construct BM chimeric mice, 6-week-old C57BL/6J WT recipient mice were exposed to γ irradiation at a dose of 950 rad. BM cells obtained from 8- to 10-week-old C57BL/6J WT or GAL3-KO donors were transferred intravenously to recipients (5 × 10^6^ cells per recipient) 2 h after irradiation. After 6 weeks of reconstitution, the chimeric mice were subjected to tMCAO, as described above. The efficiency of chimerism in recipient mice was more than 90%, according to flow cytometry analysis of blood macrophages.

### Macrophage depletion and adoptive transfer.

Recipient WT or Rag1-KO mice received intravenous injections of clodronate liposomes (Liposoma, 200 μL/mouse/day) to deplete circulating monocytes and macrophages. Forty-eight hours later, the mice were subjected to tMCAO. BMDMs (2 × 10^6^ cells per mouse) from either WT or GAL3-KO mice were transferred intravenously into the recipients immediately following reperfusion.

### Behavioral tests.

We used a battery of established behavioral tests that are highly sensitive to functional deficits after stroke as we previously described (21). For the rotarod test, the time at which a mouse fell off a rotating drum was recorded as the latency to fall. For the foot fault test, the number of errors (when the animals misplaced a forelimb such that it fell through a grid floor) was recorded for a 2 min observation period. In the learning phase of the Morris water maze test, the time spent reaching the platform was recorded to reflect spatial learning. In the memory test, time spent in the goal quadrant (where the platform was previously located) in a single 60-second probe trial was recorded to reflect spatial memory.

### MRI.

T2-weighted images were acquired and quantified by a blinded observer as previously described (52).

### Determination of infarct volume and tissue atrophy.

Six evenly spaced coronal brain sections (25 μm thick) encompassing the MCA territory were analyzed. The sections were stained with antibodies against MAP2, (Sigma-Aldrich), a neuron-specific marker, or were stained with Nissl or 2,3,5-triphenyltetrazolium chloride (TTC) stain for infarct volume assessment. The infarct volume or tissue atrophy was measured using the following formula: infarct volume = (area of contralateral brain section – area of ipsilateral noninfarcted brain section) × distance between sections. All imaging and image analysis were conducted by investigators blinded to the experimental groups.

### Flow cytometry.

Brains were dissected, and the ipsilateral and contralateral hemispheres were collected. Flow cytometry analysis was performed as previously described (21).

### Primary cortical neuronal culture and OGD.

Primary cortical neuronal cultures were prepared from E17 C57BL6/J mice as we previously described (52). For OGD, cortical neuronal cultures were placed in an incubator chamber containing 94% nitrogen, 5% CO_2_, and 1% O_2_ for 60 min. Control cultures were incubated for the same period at 37°C in humidified 95% air and 5% CO_2_.

### BMDM-neuron cocultures.

Cocultures of primary neurons and BMDMs were grown in a 24-well transwell system with a 0.4 μm pore size (Millipore Sigma). Primary neurons (2.5 × 10^5^ per well) were seeded in the lower compartment of a transwell system and subjected to 60 min OGD. BMDMs (2.5 × 10^4^ per insert) were seeded in the inserts and cultured in a separate 24-well plate. BMDMs were treated with brain lysates and other treatments for 6 h. BMDMs in transwell inserts were then washed, transferred to the neuron culture plates, and cocultured for 18 h.

### Preparation of brain lysates.

Brain lysates were prepared from brain tissue as previously described (24). Briefly, the mice were perfused transcardially with PBS. The brain hemisphere was removed, homogenized with RPMI-1640, and centrifuged at 1,000*g* for 5 min. The supernatant was diluted to 6 mL with RPMI-1640 and used as brain lysate. Two parts of brain lysate were applied to the culture system for every 8 parts of BMDM culture medium for the indicated period.

### Total protein extraction from cell culture media.

Cell culture media were first centrifuged at 1,000*g* for 5 min. The resulted supernatants (500 μL) were precipitated with 500 μL of methanol and 125 μL of chloroform. The resulting samples were then solubilized with 1× loading buffer for Western blotting.

### Preparation of lysosome-enriched lysates from BMDMs.

Enriched lysosome fractions from BMDMs were isolated with differential centrifugation as described (53). Briefly, BMDMs were harvested from 6-well plates. Cell pellets were resuspended in 500 μL of hypotonic buffer (50 mM HEPES, pH 7.4, 1 mM EDTA, and protease inhibitor cocktail) and incubated for 5 min on ice. Digitonin (Millipore Sigma) stock solution was then added to each sample to a final concentration of 0.02% to permeabilize cell membranes followed by addition of 2× isotonic buffer (100 mM HEPES, pH 7.4, 1.2 M d-sorbitol, 2 mM EDTA, and protease inhibitor cocktail). Samples were then centrifuged at 700*g* for 10 min at 4°C. Supernatants were further centrifuged at 15,000*g* for 15 min at 4°C to pellet the lysosome-enriched fraction. The enriched lysosomal pellets were solubilized in 1× loading buffer for Western blotting. The pellets obtained from the 700*g* centrifugation were combined with the supernatants from the 15,000*g* centrifugation as the remaining nonlysosomal components for Western blotting.

### Preparation of cytosolic lysates from BMDMs.

BMDMs were harvested and washed once with ice-cold 1× PBS. Cell pellets were then resuspended in 50 μL of cytosol extraction buffer (250 mM sucrose, 20 mM HEPES, pH 7.4, 10 mM KCl, 1.5 mM MgCl_2_, 1 mM EDTA, and 0.005% digitonin) supplemented with 1 mM DTT and 1× protease inhibitor cocktail, and put on ice for 5 min. The resulting samples were centrifuged at 15,000*g* for 10 min at 4°C. The supernatants were collected as cytosolic fraction.

### Western blotting.

Western blots were performed using standard SDS-PAGE. PVDF membranes were incubated in blocking buffer (LI-COR Biosciences) for 1 h at room temperature and then incubated with primary antibodies at 4°C overnight. The membranes were incubated with secondary antibodies for 1 h at room temperature in the dark (1:10,000, LI-COR Biosciences) and scanned with the LI-COR Odyssey Infrared Imaging System 9201-550U (LI-COR Biotechnology). The results were normalized to β-actin expression.

### Magic Red cathepsin B activity assay.

BMDMs were cultured in 24-well plates and treated with or without brain lysates, along with designated treatments for 40 min, 60 min, or 6 h. At the termination of treatments, cells were incubated with Magic Red cathepsin B reagent (Immunochemistry Technologies) at 37 °C for 15 min. The resulting samples were washed with PBS and evaluated by fluorescence microscopy.

### Immunofluorescence staining and image analysis.

Immunohistochemistry was performed on 25 μm free-floating brain sections. The EVOS M7000 imaging system (Invitrogen) and Nikon A1 confocal microscope were used to capture images. Quantification was performed using Fiji (v.3) and ImageJ (NIH) software by 2 investigators blinded to experimental groupings. The border of the infarct was determined by the loss of MAP2 staining and accumulation of IBA1/CD68^+^ microglia/macrophages. The stroke peri-infarct area was defined as the region that covers a radial distance of approximately 300–400 μm from the border of the infarct. For each mouse brain section, 3 randomly selected microscopic fields in the peri-infarct area were analyzed. Double immunostaining analysis was performed within the regions of interest using the Analyze Particles function in Fiji. The total number of positive cells was divided by the sampled area of each region of interest and expressed as the number of positive cells per mm².

For primary cell cultures, images were captured from 6 biological replicates per group under consistent imaging settings. Fluorescence intensity and puncta quantification were performed using the Analyze Particles function in Fiji. Colocalization analysis was conducted using the JACoP plug-in with the Pearson’s correlation coefficient. Sholl analysis of primary neurons was conducted at 10 μm radius intervals using the Simple Neurite Tracer plug-in in ImageJ. For each experimental group, 15–25 randomly selected neurons from 3 independent experiments were analyzed.

### Statistics.

Sample sizes for animal studies were determined based on pilot studies or our published body of work. Results are presented as mean ± SD in scatter dot plots unless otherwise indicated. GraphPad Prism was used for statistical analyses. The difference in means between 2 groups for continuous variables with normal distributions was assessed by 2-tailed, unpaired Student’s *t* test. The differences in means among multiple groups were analyzed using 1- or 2-way ANOVA or its nonparametric version, the Kruskal-Wallis test. Differences in means across groups with repeated measurements over time were analyzed using repeated measures ANOVA. When the ANOVA showed significant differences, pairwise comparisons between means were tested by Bonferroni’s post hoc test. Correlations for normally distributed data were tested by 2-tailed Pearson’s correlation analyses. In all analyses, *P* < 0.05 was considered statistically significant.

### Study approval.

All animal procedures were approved by the Veterans Affairs Pittsburgh Health Care System and University of Pittsburgh Institutional Animal Care and Use Committees.

### Data availability.

The data supporting the findings of this study are available within the article and in the supplementary material. Values for all data points in graphs are reported in the Supporting Data Values file.

## Author contributions

MW, ZD, JS, QY, LL, MJ, and ZL performed in vivo experiments and data analyses. MW, ZH, and FX performed cell culture experiments and data analysis. LL performed scRNA-seq analysis. MW and XH wrote the manuscript. DJRF provided the *Lgals3*^fl/fl^ mouse line. DJRF, RKL, BR, and JC provided scientific feedback and revised the manuscript. XH conceptualized, designed, and supervised the study.

## Conflict of interest

The authors have declared that no conflict of interest exists.

## Funding support

- Merit Review Award I01 BX006534 from the US Department of Veterans Affairs, Biomedical Laboratory Research and Development Service to XH.
- American Heart Association fellowship 25POST1372422 to JS.
- Veterans Affairs Senior Research Career Scientist Award to JC.

## Supplementary Material

Supplemental data

Unedited blot and gel images

Supporting data values

## Version 1 02/17/2026

In-Press Preview

## Version 2 04/15/2026

Electronic publication
