# Supplementary material for: Galectin-3 mediates lysosome-related inflammation within monocyte-derived macrophages in a mouse model of ischemic brain injury
Source: J Clin Invest. 2026 Feb 17;136(8):e194139. doi: 10.1172/JCI194139 (PMC13078880; doi:10.1172/JCI194139)
Supplement: Supplemental data [file jci-136-194139-s290.pdf]

Supplemental Material

Galectin-3 mediates lysosome-related inflammation within monocyte-derived macrophages in a mouse model of ischemic brain injury

Miao Wang[1,2], Zhentai Huang[1,2], Zhihong Du[2], Jiajing Shan[1,2], Qing Ye[1,2], Lingxiao Lu[2], Ming Jiang[2], Fei Xu[1,2], Ziyang Liu[3], David J R Fulton[4], Rehana K. Leak[5], Babak Razani[3,6], Jun Chen[1,2], Xiaoming Hu[1,2]

[1]Geriatric Research, Education and Clinical Center, Veterans Affairs Pittsburgh Health Care System, Pittsburgh, PA, USA.
[2]Department of Neurology, School of Medicine, University of Pittsburgh, Pittsburgh, PA, USA.
[3]Department of Medicine and Vascular Medicine Institute, University of Pittsburgh School of Medicine and UPMC, Pittsburgh, PA, USA.
[4]Vascular Biology Center, Medical College of Georgia, Augusta University.
[5]Division of Pharmaceutical Sciences, Duquesne University, Pittsburgh, PA, USA
[6]Pittsburgh VA Medical Center, Pittsburgh, PA, USA.

Supplemental methods:

Transient middle cerebral artery occlusion (tMCAO) was induced in young (5-month-old) and aged (20-month-old) male mice using the intraluminal occlusion method to block the left middle cerebral artery (MCA) for 60 minutes. Mice were anesthetized with 1.5% isoflurane in a gas mixture of 30% O_2 and 68.5% N_2O. A silicone-coated nylon monofilament (Doccol Corporation) was inserted into the external carotid artery and advanced into the internal carotid artery to occlude the MCA origin for 60 minutes, followed by withdrawing the monofilament to restore blood flow. Regional cerebral blood flow (rCBF) was monitored using a 2-D laser speckle imaging system (PeriCam PSI System) to confirm successful occlusion. Mice showing a rCBF reduction of >70% from pre-MCAO baseline levels were included in subsequent analyses. Sham-operated mice underwent identical surgical procedures, except for MCA occlusion. Body temperature was maintained at 37 °C ± 0.5 °C throughout the surgery with a temperature-regulated heating pad.

Permanent distal MCAO was induced in 20-month-old male mice. After anesthetizing mice, a midline incision was made in the neck to expose the left common carotid artery (CCA). Once adequately isolated, the artery was occluded by ligation, and the skin was closed with sutures. Another skin incision was made between the left eye and the ear, and the temporal muscle was dissected using bipolar electrocautery (Bipolar Coagulator, Codman & Shurtleff Inc.). A burr hole was created, followed by a craniotomy to expose the distal part of the MCA. The dura mater was incised, and the distal MCAO (dMCAO) was achieved using low-intensity bipolar electrocautery at the lateral edge of the rhinal fissure. rCBF was measured using laser Doppler flowmetry. Mice showing an rCBF reduction of >70% from pre-MCAO baseline levels were included in the study. Sham-operated animals underwent identical anesthesia and surgical procedures but were not subjected to CCA occlusion or dMCAO.

MRI scanning and analyses T2-weighted images were acquired and quantified by a blinded observer. Mice were anesthetized through a nose cone with 1-2% isoflurane delivered in Air/O_2 (2:1). The mice were then positioned on an animal bed and placed in the scanner. A rectal temperature probe was used to monitor body temperature, which was maintained at 37.0 ± 0.5˚C using a warm air heating system. Respiration was monitored (SA Instruments). MRI was performed on a 9.4T/30-cm AVIII HD spectrometer (Bruker Biospin) equipped with a 12 cm high-performance gradient set, using an 86 mm quadrature RF transmit volume coil, a 2-channel receive surface RF coil, and Paravision 6.0.1. A T2-weighted RARE sequence was used, with the following parameters: repetition time (TR)/echo time (TE) = 4000/40 ms, field of view (FOV) = 20 × 20 mm, acquisition matrix = 256 × 256, 21 slices with a slice thickness of 0.5 mm, 4 averages, and a RARE factor = 8. The infarct volume was determined by manual segmentation using Fiji (Image J) software. Brain infarct was identified by means of high signal on the T2 images. Brain infarct was calculated as (contralateral hemisphere area – ipsilateral hemisphere normal tissue area) × distance between each section. Brain edema was calculated based on the following equation: (ipsilateral hemisphere area – contralateral hemisphere area) × distance between sections.

The rotarod test Mice ran on a rotating drum (IITC Life Science Inc.) with speeds starting at 4 rpm and accelerating to 40 rpm within 300 seconds. Three consecutive trials were conducted for each mouse, with an interval of 15 minutes. The time at which a mouse fell off the drum was recorded as the latency to fall. Data was expressed as mean values from three trials per day.

Footfault test Each mouse was placed on a stainless-steel grid floor (20 cm × 40 cm with a mesh size of 4 cm^2) elevated 1 m above the floor and videotaped. The number of errors (when the animals misplaced a forelimb such that it fell through the grid) was recorded for a 2-minute-long observation period.

Morris water maze test Cognitive function was analyzed using the Morris water maze test. A square platform (11 × 11 cm^2) was submerged 2 cm beneath the water surface in a circular pool (diameter =10^9 cm) filled with opaque water. Mice were placed into the pool from one of the four locations and allowed to locate the hidden platform for 60 seconds. Each mouse was trained on 3 trials (with randomly assigned starting positions) per day to locate the platform for three consecutive days before tMCAO. At the end of each trial, the mouse was placed on the platform or allowed to stay on the platform for 30s with prominent spatial cues displayed around the room. Trials were recorded with AnyMAZE system (Stoelting Co.). In the learning phase, three trials were performed on each day. The time spent reaching the platform was recorded to reflect spatial learning. In the memory test, the platform was removed and a single 60 second probe trial was conducted. Time spent in the goal quadrant (where the platform was

previously located) was recorded to reflect spatial memory.

Flow cytometry Animals were euthanized and perfused with cold saline. Brains were dissected and the ipsilateral and contralateral hemispheres were collected. Brain homogenates were prepared with the Neural Tissue Dissociation Kit (T) using a gentle MACS dissociator with heaters (Miltenyi Biotec) following the manufacturer's instructions. The suspension was passed through a 70-µm cell strainer (Thermo Fisher) and resuspended in 30% Percoll. Single cell suspensions were separated from myelin and debris by centrifugation (800 × g, 30 min, 18°C) on a 30~70% Percoll gradient. Cells at the interface were collected and washed with Hank's balanced salt solution (HBSS; Sigma-Aldrich) containing 1% fetal bovine serum (Millipore Sigma) and 2 mM EDTA (Millipore Sigma). Brain cells were stained with fluorophore-labeled antibodies. Flow cytometric analysis was performed using an LSRII flow cytometer (BD Biosciences) and data were analyzed with FlowJo software.

Bone marrow isolation Bone marrow (BM) was isolated from the femur and tibia of WT or GAL3 KO male mice at 8-10 weeks of age. Each bone was flushed with Dulbecco's modified Eagle's medium containing 2 mM EDTA to collect BM tissues. The BM tissues were passed through a 70 µm filter, followed by centrifugation for 3 minutes at 300 rpm to collect BM cells.

The release of fluorescent dextran from lysosomes into the cytosol was assessed as described (1). Seeded BMDMs were incubated with complete media containing 100 µg/mL 10kD dextran conjugated with Alexa Fluor™ 555 and 100 µg/mL 40kD dextran conjugated with Fluorescein (Thermo Fisher) for 16h. Cells were washed twice with PBS and then treated with brain lysates in the presence or absence of TD139 for 6 h. Cells were then washed with PBS and fixed for immunostaining.

Acridine Orange (AO) Staining was performed as described (2). BMDMs were treated with brain lysates in the presence or absence of TD139 for 6 h. Cells were then washed with PBS and incubated with 1 µM AO (Sigma) for 30 min at 37 °C. The fluorescence was examined using a confocal microscope.

Primary cortical neuronal culture Cerebral cortices were isolated from E17 C57BL6/J mice and dissociated into single cells by digestion with 0.25% trypsin-EDTA (Thermo Fisher) and gentle trituration. After centrifugation, cell pellets were suspended in Neurobasal medium supplemented with B27 and 2 mM GlutaMAX (Thermo Fisher) and then seeded on poly-D-lysine coated 24 well plates. Half of the medium was replaced with fresh media every 5 d.

scRNAseq analysis Basic processing and visualization of the scRNA-seq data were performed with the Seurat package (v5.0.1) in R (v4.3.2). Briefly, low-quality cells and doublets were filtered out based on the following criteria: (i) the number of expressed genes was less than 200 or more than 7500, and (ii) the percentage of mitochondrial genes was more than 15%. The filtered count matrices underwent further normalization, and mitochondrial contamination was addressed using SCTransform function. The batch effect was removed using the Harmony R package (v1.2.0). After principal component analysis (PCA), the FindClusters function was applied to identify different clusters, followed by nonlinear dimensional reduction methods used for visualization, including uniform manifold approximation and projection (UMAP). The *Lgals3*^{high} MDMø were defined as expression level >2. The *Lgals3*^{low} MDMø were defined as expression level ≤2. For DEG analysis, genes were considered significantly dysregulated if they had a Bonferroni adjusted P-value < 0.05 and an absolute log2 fold change (log2FC) greater than 0.59. Functional enrichment analysis was performed with the online tool Metascape (http://metascape.org). All genes in the mouse genome were used as the enrichment background. After a list of DEGs was submitted, enrichment was carried out based on Kyoto Encyclopedia of Genes and Genomes (KEGG) database, with a minimum count of 3 and an enrichment factor (the ratio between the observed counts and the counts expected by chance) larger than 1.5. Metascape then returned a list of significantly over-represented (p-value < 0.01) KEGG terms.

Two single-cell RNA sequencing (scRNA-seq) datasets were downloaded from the Gene Expression Omnibus (GEO) database, including expression matrix of the CD45^{intermediate} and CD45^{high} immune cells and CD45^{negative}/Ly6c^{high} endothelia cells isolated from mice 2d and 14d after tMCAO or sham surgery and the expression matrix of the peripheral bold leukocytes from mice 2d and 14d after tMCAO (GSE225948).

Supplemental Figures:

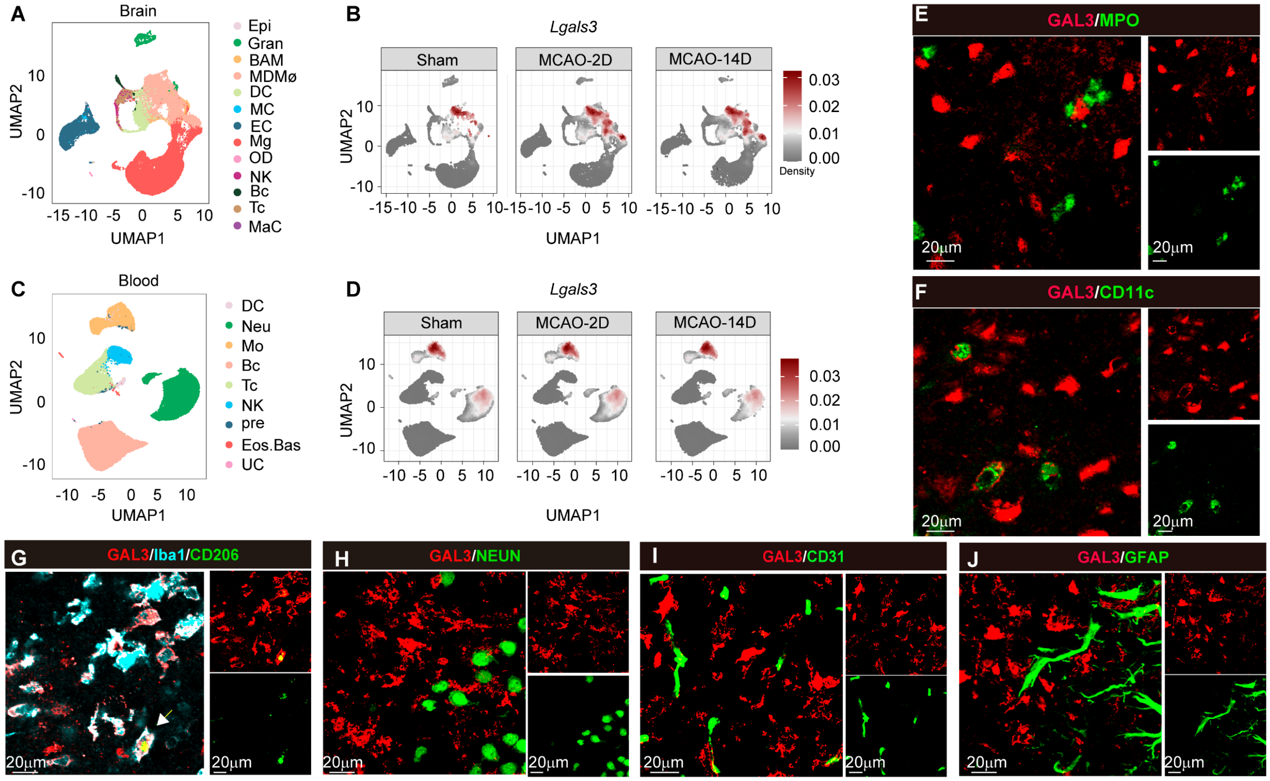

Figure S1. GAL3 is highly expressed in MDMø and peripheral monocytes after ischemic stroke. (A) UMAP plot showing clustering and cluster annotations of brain cells from young male mice 2d and 14d after tMCAO or sham operation. **(B)** Density plots in the UMAP space showing the expression of *Lgals3* in the brain cells 2d and 14d post-tMCAO and after sham operation. **(C)** UMAP plot showing clustering and cluster annotations of blood cells from young male mice 2d and 14d after tMCAO or sham operation. **(D)** Density plots in the UMAP space showing the expression of *Lgals3* in the blood cells 2d and 14d post-tMCAO or sham operation. Scale bar represents densities based on kernel density estimation of gene expression. BAM–Border-associated Mø, Bc–B cells, DC–dendritic cells, EC–endothelial cells, Epi–epithelia-like cells, Eos.Bas–Eosinophil.Basophils, Gran–granulocytes, MaC–mast cells, MC–mural cells, Mg–microglia, MDMø–monocyte derived macrophages, Mo–monocytes, Neu–Neutrophil, NK–natural killer cells, Tc–T cells, OD–oligodendrocytes, pre–hematopoietic precursors, UC–unclassified. **(E-F)** Co-immunostaining of GAL3 with MPO^{+} neutrophil (E) or CD11c^{+} dendritic cells (F) in peri-infarct areas 5d after tMCAO. **(G)** Co-immunostaining of GAL3 with IBA1^{+}CD206^{+} BAM in peri-infarct areas 5d after tMCAO. White arrow indicates GAL3^{+}IBA1^{+}CD206^{+} BAM. **(H-J)** Co-immunostaining of GAL3 with NEUN^{+} (H), CD31^{+} (I), or GFAP^{+} (J) cells in peri-infarct areas 5d after tMCAO. (related to Figure 1)

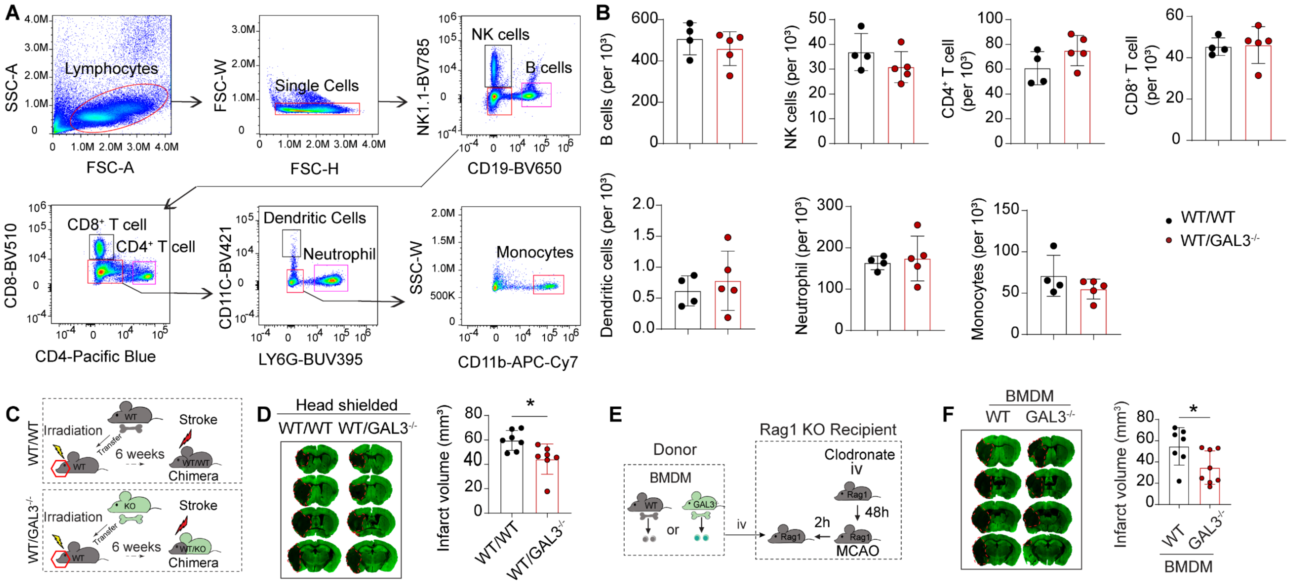

Figure S2. WT/GAL3 KO bone marrow chimera mice and Rag1 KO mice that received GAL3 KO BMDM exhibit smaller infarct 3d after tMCAO compared to respective control mice. (A-B) Flow cytometric analysis of immune cell composition in blood collected from WT/GAL3$^{-/-}$ and WT/WT chimera mice 3d after tMCAO. **(A)** Gating strategy. **(B)** Quantification of various immune cells in the blood. N = 4-5/group. **(C)** Experimental design. The bone marrow of GAL3 KO or WT mice was transferred to irradiated WT recipients (head shielded). **(D)** WT/GAL3$^{-/-}$ and WT/WT chimera mice were subjected to 60 min tMCAO. Brain infarct was measured by MAP2 staining 3d after tMCAO. N = 7/group. **(E)** Experimental design of monocyte depletion and BMDM adoptive transfer in Rag1$^{-/-}$ mice. **(F)** Brain infarcts were measured in WT or GAL3$^{-/-}$ BMDM transferred Rag1$^{-/-}$ mice by MAP2 staining 3d after tMCAO. N = 7-8/group. *p<0.05. Two-tailed, unpaired Student's *t* test. (related to Figure 3)

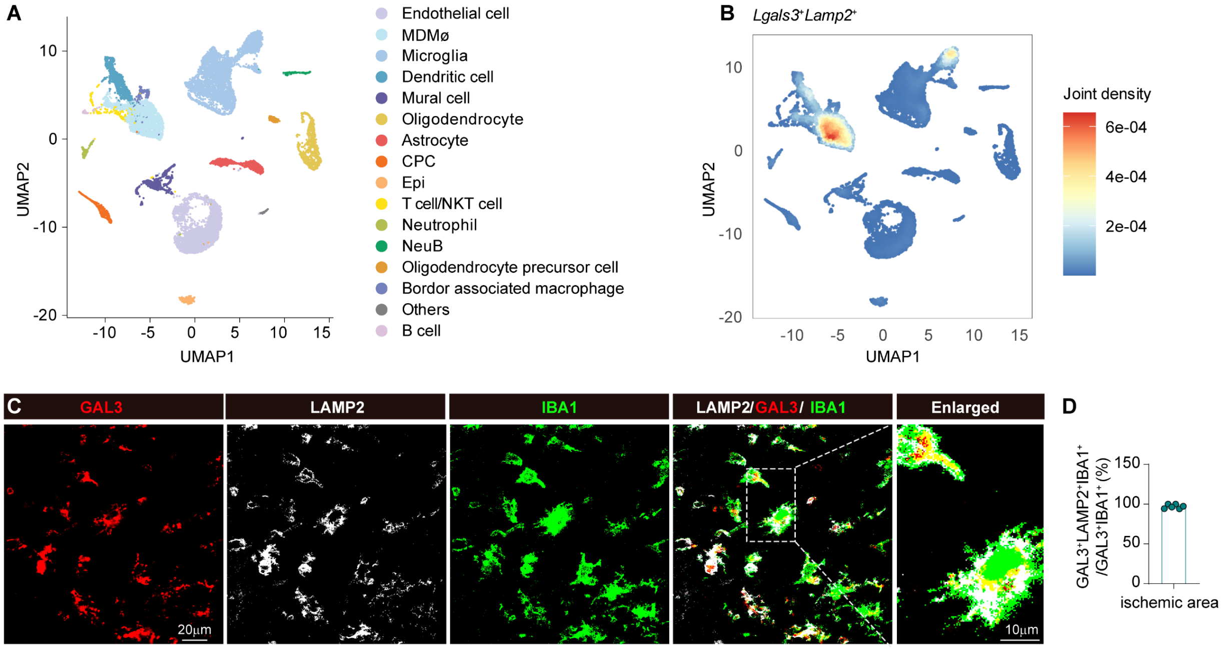

Figure S3. GAL3 accumulates in lysosomes in MDMø early after tMCAO. (A) UMAP plot showing clustering and cluster annotations of brain cells from young male mice 3d after tMCAO or after sham operation. **(B)** UMAP plot showing joint density of *Lgals3*$^+$*Lamp2*$^+$ cells. **(C)** Representative LAMP2/GAL3/IBA1 staining in ischemic brains 3d after tMCAO. **(D)** The percentage of GAL3$^+$LAMP2$^+$IBA1$^+$ cells amongst all GAL3$^+$IBA1$^+$ cells. N = 6/group. (related to Figure 4)

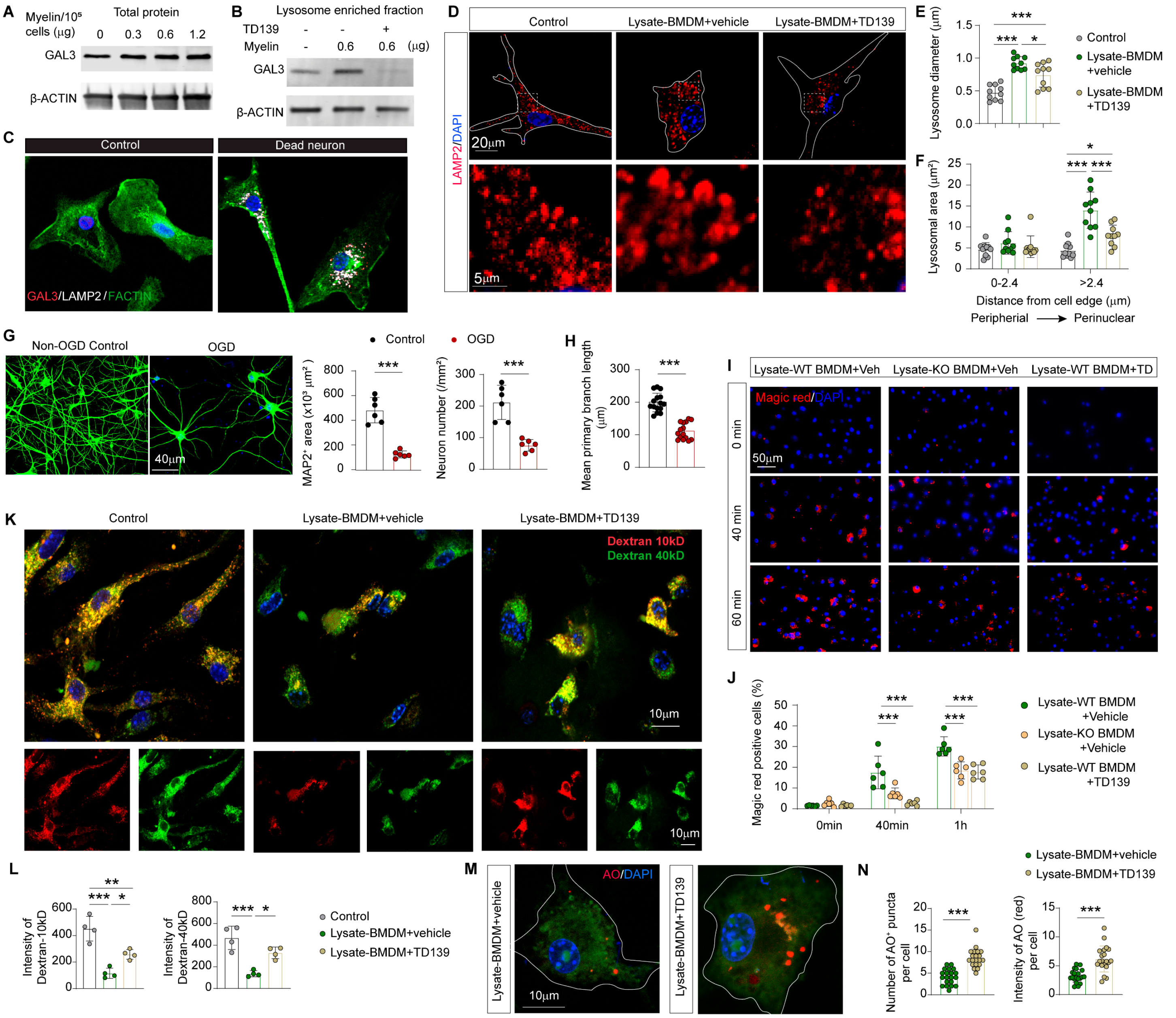

Figure S4. Brain lysate-induced GAL3 expression in BMDMs changes lysosomal morphology, neurotoxicity, cathepsin activity, and lysosomal membrane permeability. (A) Immunoblot of total GAL3 expression after different doses of myelin fragment treatment in cultured bone marrow-derived macrophages (BMDMs). **(B)** Immunoblot of GAL3 expression in lysosome-enriched fraction after treatment with 0.6 µg myelin fragment with or without TD139 (10 µM). **(C)** Representative images of GAL3, LAMP2, F actin, and nuclear DAPI staining in control and dead neuron-treated BMDMs. **(D)** Representative images of LAMP2 and nuclear DAPI staining in control and brain lysate-treated BMDMs with or without TD139 (10 µM). **(E)** Quantification of lysosomal diameters. **(F)** Quantification of lysosomal areas in different groups of cells. N = 10 randomly selected cells per condition from three independent experiments. **(G)** Primary cultured neurons in 24-well plates were exposed to 60 min of oxygen glucose deprivation (OGD). Representative images show MAP2 neuronal immunostaining. The coverage areas of MAP2-stained neurons and the numbers of $MAP2^+$ neurons were quantified. N = 6 samples per condition. **(H)** Sholl analysis shows mean primary branch length. N = 15 randomly selected neurons from 3 independent experiments. **(I)** Cathepsin B activity was analyzed by Magic Red staining in brain lysate-treated WT BMDMs with vehicle or TD139 (10 µM) treatment, or GAL3 KO BMDMs. Blue shows nuclear DAPI staining. **(J)** Quantitative analysis of the percentages of Magic Red positive cells among total cells. N = 6 samples per condition. **(K-L)** BMDMs were preloaded with dextran of different sizes and

fluorochromes (Alexa Fluor™ 555–conjugated 10kD dextran and Fluorescein-conjugated 40kD dextran) for 16h. Cells were then treated with brain lysate with vehicle or TD139 (10 μM) for 6h. N = 4 samples per condition. **(K)** Representative images showing individual channels (10- and 40 kD Dextrans) and overlays under different treatment conditions. **(L)** The fluorescence intensities of Dextran 10kD and Dextran 40kD were quantified. **(M-N)** BMDM were treated with brain lysate together with vehicle or TD139 (10 μM) for 6h. Cells were stained with acridine orange (AO) and imaged 30 min after AO staining. **(M)** Representative images showing AO-labeled red lysosomal puncta within BMDMs. Green shows AO staining in cytosol under neutral pH. Blue shows nuclear DAPI staining. **(N)** The numbers of AO-labeled red puncta and the intensity of AO staining were quantified per cell. N = 20 randomly selected cells per condition from two independent experiments. Data are mean ± SD. *p<0.05, **p<0.01, ***p<0.001. Two-tailed, unpaired Student's *t* test (G, H, N) or one-way ANOVA & Bonferroni (E, F, J, L). (related to Figure 4-6)

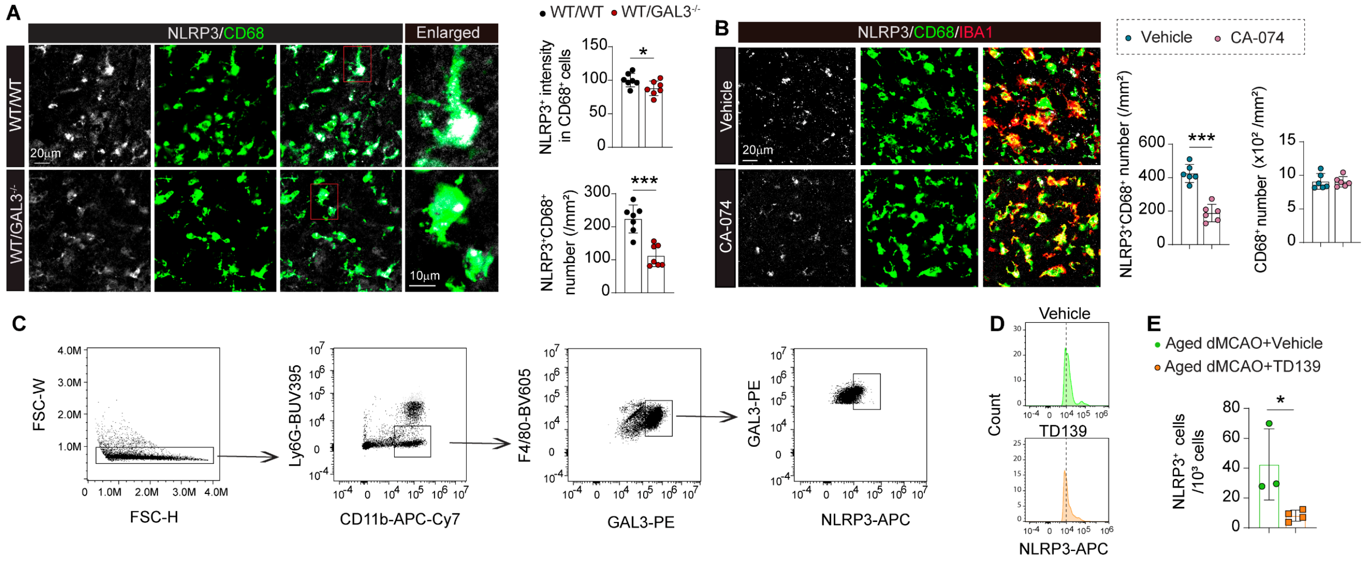

Figure S5. GAL3-mediated cathepsin induction enhances NLRP3 expression in MDMø. (A) Immunostaining of NLRP3 and CD68 in brain from WT/WT and WT/GAL3$^{-/-}$ chimeric mice 3d following tMCAO. NLRP3 intensity within CD68^{+} cells and number of NLRP3^{+}CD68^{+} cells were quantified. N = 7/group. **(B)** Immunostaining of NLRP3, CD68, and IBA1 in WT mice treated with vehicle or CA-074 (10 mg/kg, iv, 2h after tMCAO and then daily for 2d) 3d following tMCAO. Number of NLRP3^{+}CD68^{+} cells and CD68^{+} cells were quantified. N = 6/group. **(C-E)** Aged male mice were subjected to dMCAO and treated with vehicle or TD139 (0.4 mg/kg, starting from 2h after dMCAO then daily for 2d). Flow cytometry detected the number of NLRP3^{+}GAL3^{+} monocytes in the blood 3d after dMCAO. **(C)** Gating strategy for NLRP3^{+}GAL3^{+}CD11b^{+}F4/80^{+}LY6G^{-} monocytes. **(D)** Histogram showing the expression of NLRP3 in GAL3^{+}CD11b^{+}F4/80^{+}LY6G^{-} monocytes. **(E)** Quantification of the number of NLRP3^{+}GAL3^{+}CD11b^{+}F4/80^{+}LY6G^{-} monocytes in the blood. N = 3-4/group. Data are plotted as mean ± SD. *$p<0.05$, ***$p<0.001$. Two-tailed, unpaired Student's *t* test. (related to Figure 7)

References:

1. Zarnegar B, Carow B, Eriksson J, Spennare E, Ohlund P, Akpinar E, et al. Matrix-M adjuvant triggers inflammasome activation and enables antigen cross-presentation through induction of lysosomal membrane permeabilization. *NPJ Vaccines.* 2025;10(1):184.
2. Hu P, Wang J, Qing Y, Li H, Sun W, Yu X, et al. FV-429 induces autophagy blockage and lysosome-dependent cell death of T-cell malignancies via lysosomal dysregulation. *Cell Death Dis.* 2021;12(1):80.
