# Supplementary material for: Galectin-3 mediates lysosome-related inflammation within monocyte-derived macrophages in a mouse model of ischemic brain injury
Source: J Clin Invest. 2026 Feb 17;136(8):e194139. doi: 10.1172/JCI194139 (PMC13078880; doi:10.1172/JCI194139)
Supplement: Unedited blot and gel images [file jci-136-194139-s291.pdf]

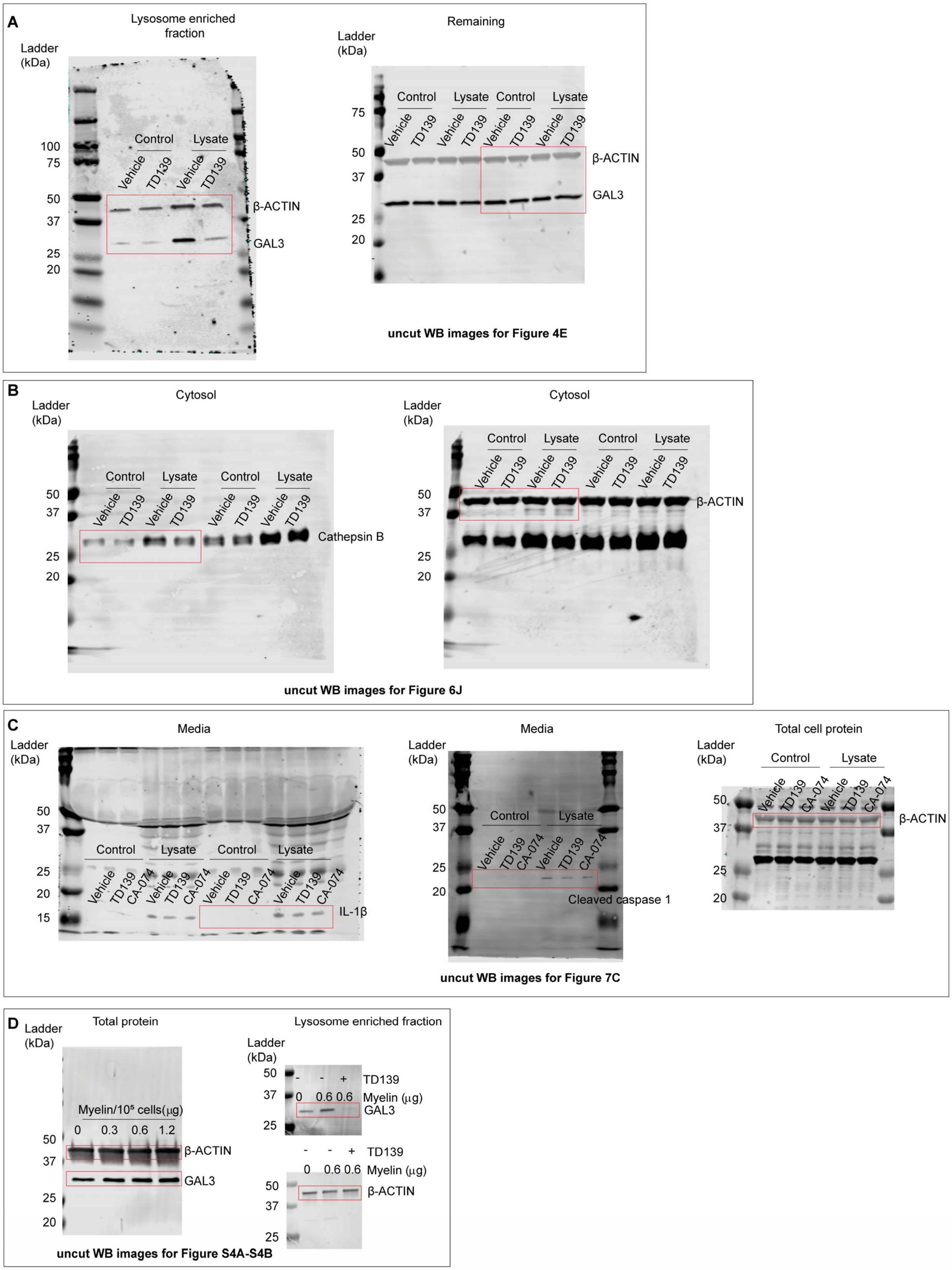

Whole gel scanning for western blotting analyses. (A) Full-length blot scan for western blotting analyses of GAL3 and β-ACTIN in lysosome enriched fraction and remaining fraction from control or brain lysate-treated BMDMs in Figure 4E. **(B)** Full-length blot scan for western blotting analyses of cathepsin B and β-ACTIN in cytosolic fraction from control or brain lysate-treated BMDMs in Figure

6J. **(C)** Full-length blot scan for western blotting analyses of IL-1β and cleaved caspase 1 in conditioned media, and β-ACTIN in the corresponding cells from control or brain lysate-treated BMDM culture in Figure 7C. **(D)** Full-length blot scan for western blotting analyses of GAL3 and β-ACTIN in total cell lysates or lysosome enriched fraction from control or myelin-treated BMDMs in Figure S4A-B. (related to Figure 4, 6, 7, and S4)
